# Insight into the global evolution of Rodentia associated *Morbilli*-*related* paramyxoviruses

**DOI:** 10.1038/s41598-017-02206-0

**Published:** 2017-05-16

**Authors:** Wissem Ghawar, Hervé Pascalis, Jihéne Bettaieb, Julien Mélade, Adel Gharbi, Mohamed Ali Snoussi, Dhafer Laouini, Steven M. Goodman, Afif Ben Salah, Koussay Dellagi

**Affiliations:** 1 Centre de Recherche et de Veille sur les maladies émergentes dans l’Océan Indien (CRVOI), Plateforme de Recherche CYROI, Sainte Clotilde, La Réunion France; 2 Laboratory of Medical Epidemiology, Institut Pasteur de Tunis (IPT), Tunis-Belvédère, Tunis Tunisia; 3 Laboratory of Transmission, Control and Immunobiology of Infections (LTCII), LR11IPT02, Institut Pasteur de Tunis (IPT), Tunis-Belvédère, Tunis Tunisia; 4 Université Tunis El Manar, Tunis, Tunisia; 5 Université de La Réunion, UMR PIMIT “Processus Infectieux en Milieu Insulaire Tropical”, INSERM U1187, CNRS 9192, IRD 249, Plateforme de Recherche CYROI, Saint Denis, La Réunion France; 6 Field Museum of Natural History, 1400 S. Lake Shore Dr, Chicago, IL 60605-2496 USA; 7 Association Vahatra, BP 3972, Antananarivo, 101 Madagascar

## Abstract

One portion of the family *Paramyxoviridae* is a group of *Unclassified Morbilli*-*Related Viruses* (*UMRV*) recently recognized in wild small mammals. At a global level, the evolutionary history of these viruses is not properly understood and the relationships between *UMRV* and their hosts still remain largely unstudied. The present study revealed, for the first time, that Rodentia associated *UMRV* emerged from a common ancestor in southern Africa more than 4000 years ago. Sequenced *UMRV* originating from different regions in the world, clustered into four well-supported viral lineages, which suggest that strain diversification occurred during host dispersal and associated exchanges, with purifying selection pressure as the principal evolutionary force. In addition, multi-introductions on different continents and islands of Rodentia associated *UMRV* and spillover between rodent species, most probably *Rattus rattus*, were detected and indicate that these animals are implicated in the vectoring and in the worldwide emergence of this virus group. The natural history and the evolution dynamics of these zoonotic viruses, originating from and hosted by wild animals, are most likely shaped by commensalism related to human activities.

## Introduction

Paramyxoviruses (PV) belong to a family of negative-sense single-stranded RNA viruses that infect a large range of hosts including mammals, birds, reptiles and fish^1^. The family includes known pathogens of humans (e.g. *Measles* and *Mumps viruses*), rodents (e.g. *Sendai virus*), birds (e.g. *Newcastle Disease virus*), livestock (e.g. *Rinderpest virus*), canids (e.g. *Canine Distemper virus*) and new emerging zoonotic viruses of medical importance such as *Hendra* or *Nipah viruses* ^1, 2^. Some other PV may have significant socio-economic impact such as the equine disease outbreak caused by *Salem virus* ^3^. Recently, novel and very diverse PV, tentatively named as *Unclassified Morbilli*-*Related Viruses* (*UMRV*, *Paramyxovirinae*, *Paramyxoviridae*) were reported to infect at high rates different orders of wild small mammals, in different geographical areas, including Rodentia, Chiroptera and Afrotheria^4, 5^. To date, a few *UMRV* have been reported to be pathogenic to their animal hosts, such as the *J* and *Belinga viruses* inducing hemorrhagic lesions in rodents and bats, respectively^6, 7^, but for most PV, little is known about their pathogenicity, origin and evolution.

The different recognized *UMRV* are phylogenetically closely related, but their evolutionary history is still poorly understood. In a previous study^5^, we have characterized the existence of spillover events for these viruses between different orders of animal hosts, including introduced populations of *Rattus*. Furthermore, we underlined that there was probably viral flow between some southwestern Indian Ocean (SWIO) islands and Africa. However, from a global perspective it was not determined the origin of these exchanges or direction of flow between different geographical areas. In another study of *UMRV* transmission amongst Malagasy bats^8^, the macro-evolutionary mechanisms underlying genetic diversification in these viruses was unraveled and host-switching was shown to be the main process generating viral genetic diversity. Hence, by extrapolation, introduced rodents, such as *Rattus*, might act as epidemiological bridges between *UMRV* and native mammal species.

Currently available *UMRV* sequences were obtained principally from mammal hosts belonging to the orders Chiroptera and Afrotheria from Madagascar and to a lesser extent from Africa. Hence, to understand better Rodentia associated *UMRV*, at a broader geographical scale, particularly the role of *Rattus* as a major diffusing vector, it is necessary to investigate the evolutionary trajectories of these viruses. As rodents are the most speciose mammal order in the world and host a diversity of zoonotic pathogens^9^, eco-epidemiological studies of these animals are highly relevant to human health^10, 11^. Moreover, *UMRV* are phylogenetically closely related with the genus *Morbillivirus* and some evidence exists that the latter emerged from the former and more specifically from Rodentia paramyxoviruses^12^. However, to date, the evolutionary origins of *Morbillivirus* remain unclear.

In this study, we investigated the phylogeography and evolutionary history of *UMRV* among Rodentia reservoir species and more specifically the role *Rattus rattus* plays in the global diffusion of these viruses. We first identified new Rodentia *UMRV* from three rodent species collected in Tunisia, all indigenous to North Africa and sympatrically occurring with introduced *R*. *rattus*. Then we compared the *UMRV* sequences derived from the Tunisian samples to those previously described elsewhere in the world, including from rodent species endemic to Madagascar. Based on these comparisons and to the geographic origins of the samples, we highlight the relationships between *UMRV* and their rodent reservoir hosts, thus providing new insights into the mechanisms of their global evolution and their worldwide distribution. We also present new phylogenetic data on the origins of *UMRV* and *Morbillivirus*.

## Results

### Comparison of partial to complete *L*-*gene* sequences among PV

As only partial sequences of the *polymerase gene* have been reported for *UMRV*, in contrast to other *Paramyxoviridae* where full sequences of this gene are available in databases, we first determined if partial *L*-*gene* sequences can be used to conduct phylogenetic analyses at the level of viral species. To this end we carried out a double phylogenetic analysis using a representative set of the *Paramyxoviridae* containing 179 different sequences and comparison of the associated phylogenetic tree to the one derived from the analysis of the polymerase locus across its full length (8373 base pairs). As shown in Supplementary Fig. S1, the two trees mirror each other with regards to the topology of viral genera, generally with significant Posterior Bayesian values, and are notably concordant at the level of viral species (outlined in color). Only seven topological inconsistencies were found (each identified in the figure by a red asterisk), representing less than 4% of the total number of analyzed sequences (n = 7/179). A description of the analysis and associated statistical analyses associated with the phylogenetic signals are given in Supplementary Text S1 and Supplementary Table S1. Considering these results, the use of partial *L*-*gene* sequences appeared as a viable alternative for *Paramyxoviridae* phylogenetic reconstruction.

### PV infecting wild rodents from Tunisia belongs to *UMRV*

Rodents belonging to four species, including three of the family Muridae (*Psammomys obesus*, *Meriones shawi*, *Rattus rattus*) and one of the family Ctenodactylidae (*Ctenodactylus gundi*), were randomly selected (40 individuals per species) from a large panel of animals captured in Tunisia and tested by RT-PCR for infection with PV (Supplementary Table S2). PV RNAs were detected in 49 out of 160 rodents, yielding a global positivity rate of 30.6% (range 2.5–50% according to the species, *p* < 10^−3^, Table 1). There was no significant difference in the infection rates of the hosts based on sex, morphometric variables and sampling site location.

**Table 1:** Detection by RT-PCR of PV infection in rodents from Tunisia based on sampling site and different biological parameters.

	*Psammomys obesus*	*Meriones shawi*	*Rattus rattus* ^1^	*Ctenodactylus gundi*
	(n = 40)	(n = 40)	(n = 40)	(n = 40)
PV infection (%, *P*)	20	1	9	19
	(50, <10^−3^)	(2.5, <10^−3^)	(22.5, <10^−3^)	(47.5, <10^−3^)
**Based on capture sites** (**n** (**%**))				
Sidi Bouzid	20 (100)	1 (100)	9 (100)	11 (57.9)
Tataouine	/	/	/	8 (42.1)
*P*	/	/	/	NS
**According to gender** (**n** (**%**))				
Male	14 (70)	1 (100)	6 (66.7)	8 (42.1)
Female	6 (30)	/	3 (33.3)	11 (57.9)
*P*	NS	/	NS	NS
**According to morphometric parameters** (**Mean** ± **SD** (***P***))				
Weight (g)	129.70 ± 23.85 (NS)	35	96.66 ± 28.28 (NS)	227.94 ± 46.30 (NS)
Ear length (mm)	16.35 ± 1.13 (NS)	14.00	21.66 ± 0.86 (NS)	18.21 ± 1.22 (NS)
Head and body length (mm)	155.30 ± 10.08 (NS)	105.00	164.88 ± 16.72 (NS)	199.10 ± 11.65 (NS)
Tail length (mm)	125.95 ± 7.30 (NS)	152.00	199.11 ± 22.72 (NS)	28.15 ± 9.48 (NS)
Hind foot length (mm)	36.00 ± 1.37 (NS)	27.00	33.44 ± 1.58 (NS)	42.21 ± 1.58 (NS)

NS: Not significant.

^1^Introduced.

PV partial *L*-*gene* sequences were obtained from 45 PV positive rodents from Tunisia (designated hereafter as RodPV-Tun). In order to assess their evolutionary position, we performed a Bayesian phylogenetic analysis including most of the PV genera. As shown in Supplementary Fig. S2, RodPV-Tun formed a well-supported sister clade along with morbilliviruses (BP = 0.98), identifying them as novel *UMRV*. RodPV-Tun can be further subdivided into two discrete phylogroups (BP = 0.92). The first phylogroup forms a clade that includes all RodPV-Tun hosted by *P*. *obesus* and most individuals of *C*. *gundi*, as well as the unique sequence from *M*. *shawi*. The *Mossman* and *Tupaia viruses* ^13, 14^, along with *Salem virus* ^3^, occur at a basal position to this clade. The second phylogroup comprises the balance of the RodPV-Tun, specifically those detected in *R*. *rattus* and the remaining sequences from *C*. *gundi*, together with *Tailam*, *Beilong* and *J viruses* ^6, 15, 16^. In contrast to the phylogroup 1, where *P*. *obesus* and *C*. *gundi* clustered separately into two rather well supported subclades (BP = 0.67 and 1, respectively), phylogroup 2 is composed of mixed sequences from *C*. *gundi* and *R*. *rattus*.

Altogether, our sampling reveals that at least two main PV lineages are co-circulating among Tunisian rodents: *C*. *gundi* are infected by both lineages, whereas only one lineage was recovered from our samples of *P*. *obesus*, *M*. *shawi* and *R*. *rattus*. Interestingly, the viral lineage that infects *C*. *gundi* and *R*. *rattus* was only detected in these rodents, despite the sympatric occurrence of other species and only from *C*. *gundi* trapped in the Sidi Bouzid Governorate.

### Phylogeny of Rodentia associated *UMRV*

**Figure 1:**
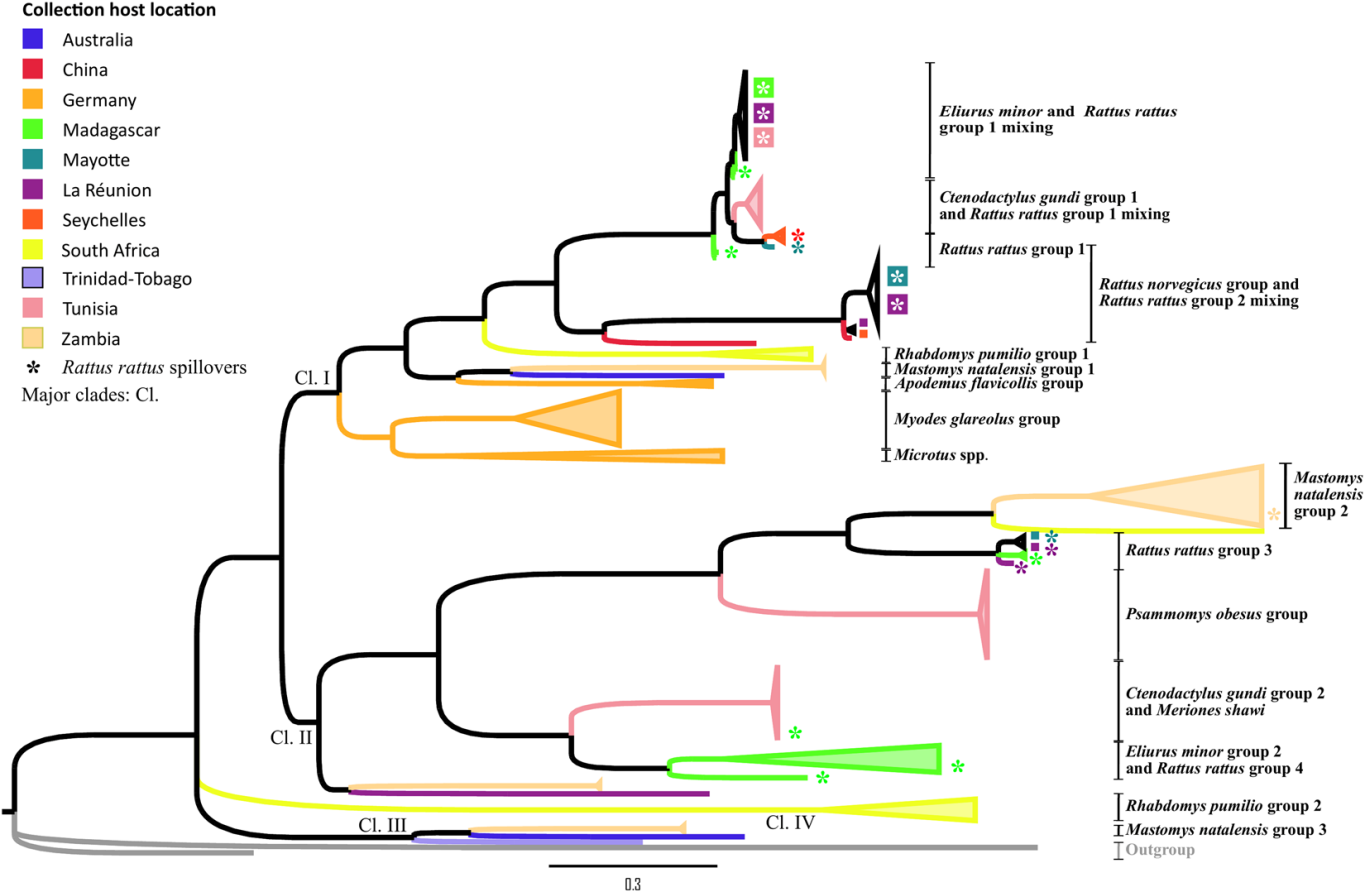
Bayesian phylogeny of the partial *L*-*gene* of Rodentia associated *UMRV* infecting Tunisian rodents and associated GenBank accession numbers. The branches are colored based on the sequence geographic origin as delineated in the figure key. Groups are identified according to the host *UMRV* species. Viruses preceded by an asterisk indicate a spillover event between *Rattus rattus* and other Rodentia species. Virus designations are as follows: Virus name or virus affiliation/host/Genbank accession number/country of origin/collection year/host family. Abbreviations are detailed in Supplementary Data S4. Two *Respirovirus* sequences (HQ660195 and AB844426) form the outgroup.

Using a larger data set downloaded from GenBank, we then extended the phylogenetic analysis of RodPV-Tun, to *UMRV* hosted by rodents from different parts of the world. Rodents included in this analysis belong to four families (Nesomyidae, Ctenodactylidae, Cricetidae and Muridae). They originated from 11 countries distributed on five continents (Africa: Tunisia, Zambia, South Africa; America: Trinidad/Tobago; Asia: China; Australia; and Europe: Germany), as well as from SWIO islands (Madagascar, Mayotte, La Réunion, Seychelles); the distribution of hosts was biased towards murids, which are disproportionately represented in GenBank. The topology of the tree defines, at the global level, four relatively well-supported major paraphyletic clades (Fig. 1 and Supplementary Fig. S3; BP = 0.78, 0.93, 1 and 1, respectively, for clades I, II, III and IV). Each clade grouped *UMRV* that were hosted by taxonomically different rodents originating from distant countries (Fig. 1, branches of the tree colored to highlight geographic origins of samples).

A critical point is that the evolutionary history of *UMRV* cannot be simply explained based on the geographical localities the samples were obtained. For example, the *UMRV* from Tunisia were genetically related to those isolated from SWIO islands. In both clades I and II, closely related-PV infect *C*. *gundi*, a species restricted to northern Africa, and *Eliurus* spp., members of a subfamily endemic to Madagascar. Likewise, *UMRV* from Germany harbored by a rodent species native to Europe and western Asia (*Apodemus flavicollis*), were closely related to those from Zambia hosted by a broadly distributed sub-Saharan rodent (*Mastomys natalensis*).

The tree topology indicates some host-virus species associations. For example, *UMRV* infecting *R*. *rattus* from SWIO islands divide into four groups clustered into two well-supported lineages. Similarly, *UMRV* infecting *Rhabdomys pumilio* from South Africa comprise two groups clustered into two well-supported lineages and those isolated from *M*. *natalensis* from Zambia form three groups clustered into different well-supported lineages. Further, some rodent hosts are the unique link to their respective viruses and infected with only a single viral lineage, such examples herein include *A*. *flavicollis*, *Myodes glareolus* and *Microtus* spp. from Germany and *P*. *obesus* from Tunisia.

The evolutionary history of *UMRV* infecting Rodentia suggests several different types of events. *UMRV* sequences that are genetically close and found in different rodent species, whether geographically close or distant, indicate the occurrence of host-jumps and/or spatial diffusion. The observation that most of these events involve introduced *Rattus* spp. (represented with an asterisk in Fig. 1 and Supplementary Fig. S3) pinpoints the role of this genus as a major diffusing vector. Tunisian *C*. *gundi* and *R*. *rattus* in clade I, as well as Tunisian *M*. *shawi* and Malagasy *R*. *rattus* in clade II, are two examples illustrating *UMRV* species jumps. In both cases, *C*. *gundi* sequences were closely associated with viruses hosted by Malagasy *Eliurus minor*, which presents a spillover with *R*. *rattus* introduced to Madagascar.

### Phylogeography analysis of Rodentia associated *UMRV*

**Figure 2:**
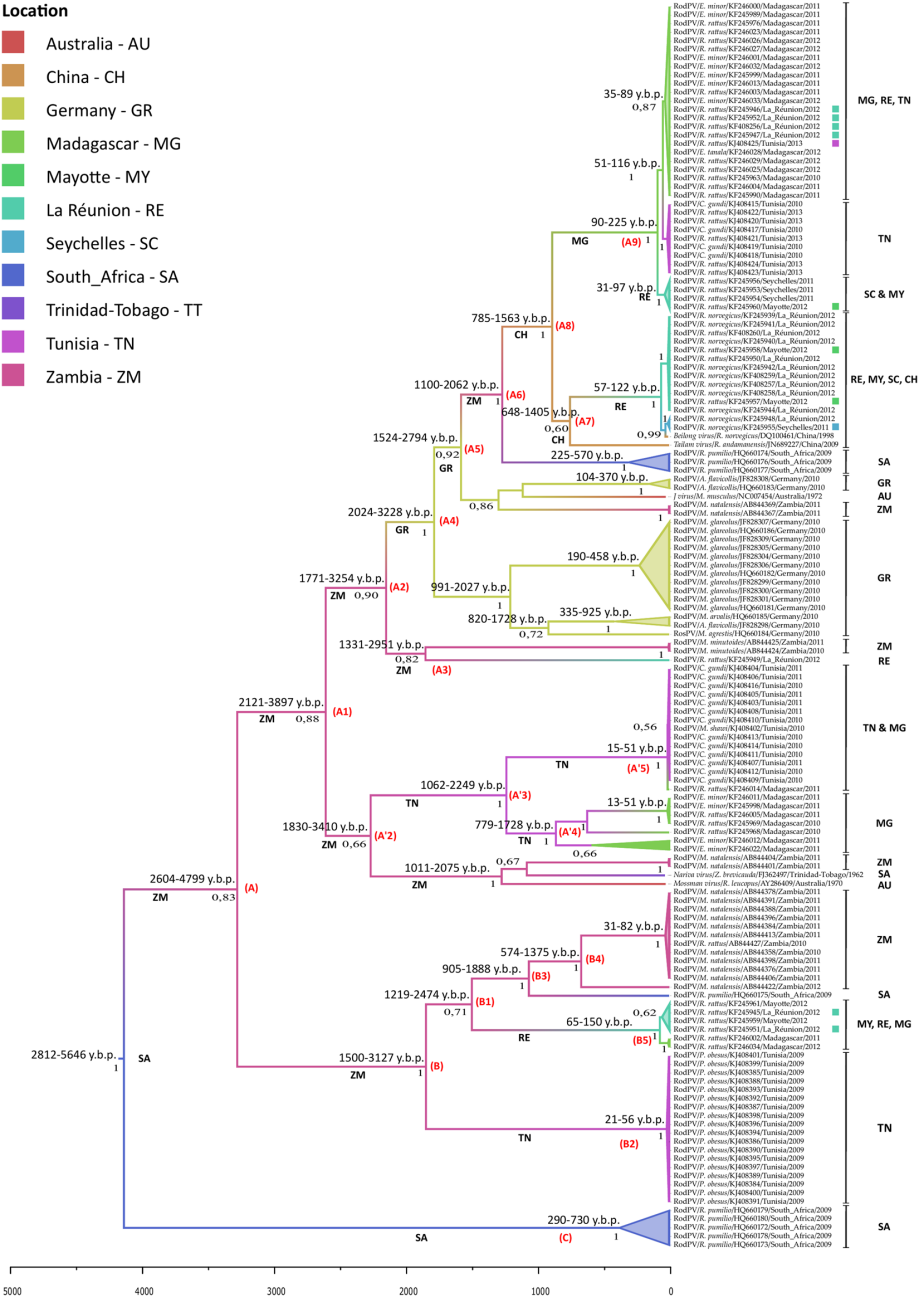
Maximum clade credibility tree of Rodentia associated *UMRV*. The phylogenetic relationships and temporal evolutionary history have been estimated based on molecular clock analysis. Branch lengths are temporally scaled, and the x-axis presents a time scale (years before present). The tree branches are colored following the most probable location of their descendent nodes as specified in the figure. Geographical groups are identified according to the origin of the *UMRV*. Viruses preceded by a small colored square indicate a spillover event between *Rattus rattus* and other Rodentia species with the color indicating the location of the spillover. Node points indicate Bayesian posterior probabilities and the 95% HPD. Virus designations are as follows: Virus name or virus affiliation/host/Genbank accession number/country of origin/collection year/host family. Abbreviations used are detailed in Supplementary Data S4.

In Fig. 2, we present the Maximum Clade Credibility (MCC) tree; the branches are colored according to the most probable location of their descendent nodes. Two important topological changes have taken place between Figs 1 and 2. In the latter, *Mus minutoides* group from clade II was sister to a sequence of *R*. *rattus* from La Réunion and all have a basal position in clade I. Indeed, a new separate phylogroup was formed by the association of a portion of clade II sequences (specifically the subgroup of *C*. *gundi* group 2 clustered with a sequence of *R*. *rattus* from Madagascar, *E*. *minor* group 2 and *R*. *rattus* group 4) clustered with clade III and all reach the clade I in the Fig. 2.

Statistical analysis of the phylogeographic tree presented in Fig. 2 indicates that it most probably is rooted in southern Africa. Based on available samples, Zambia is the most probable location for the origin of two main clades (A1 and B), and of the two major phylogroups (A2 and A′2). Hence, rodent associated *UMRV* sequences most probably had their origin in southern Africa and their currently circulating sequences emerged from a common ancestor more than 4000 years before present (y.b.p.). Based on these analyses, Rodentia associated *UMRV* occurred about 2500 y.b.p. in what is today South Africa and Zambia. The most recent common ancestor (T_MRCA_) for the lineage of all Rodentia associated *UMRV* from Australia and Trinidad/Tobago dates back to 3000 y.b.p. (clade A1; 95% HPD = 2121–3897 y.b.p.); the European Rodentia-*UMRV* (represented by the sequences from Germany) emerged more recently (clade A4; 95% HPD = 1524–2794 y.b.p.); and Asian Rodentia-*UMRV* (represented by China) was the last to diverge (clade A7; 95% HPD = 785–1563 y.b.p.). It should be noted that clades A9, A′5, B5 and B2, currently circulating on SWIO islands and in Tunisia, have a recent emergence, probably less than 200 y.b.p., with earlier introductions of Rodentia-*UMRV* on La Réunion and Madagascar dating back to around 2100 y.b.p. (clade A3; 95% HPD = 1331–2951 y.b.p.) and 1200 y.b.p. (clade A′4; 95% HPD = 779–1628 y.b.p.), respectively (Supplementary Fig. S4).

Bayesian phylogeographic analysis showed 46 well-supported route linkages between countries. We used a Bayes factor (BF) cutoff of 3.0 to determine significance (Supplementary Data S1). Most routes involved African countries and SWIO islands, suggesting that these geographical regions played an important role in the migration of rodent associated *UMRV*. The route with the strongest support was found between Zambia and Trinidad/Tobago with a BF of 207. Most routes commence in African countries (17 with eight routes from South Africa) followed by SWOI (15 routes), Trinidad & Tobago (seven routes), Australia (four routes) and Germany (three routes). On the other hand, China was found on 10 routes. The map with the rates of transitions constructed using Spread is shown as Supplementary Fig. S5.

**Figure 3:**
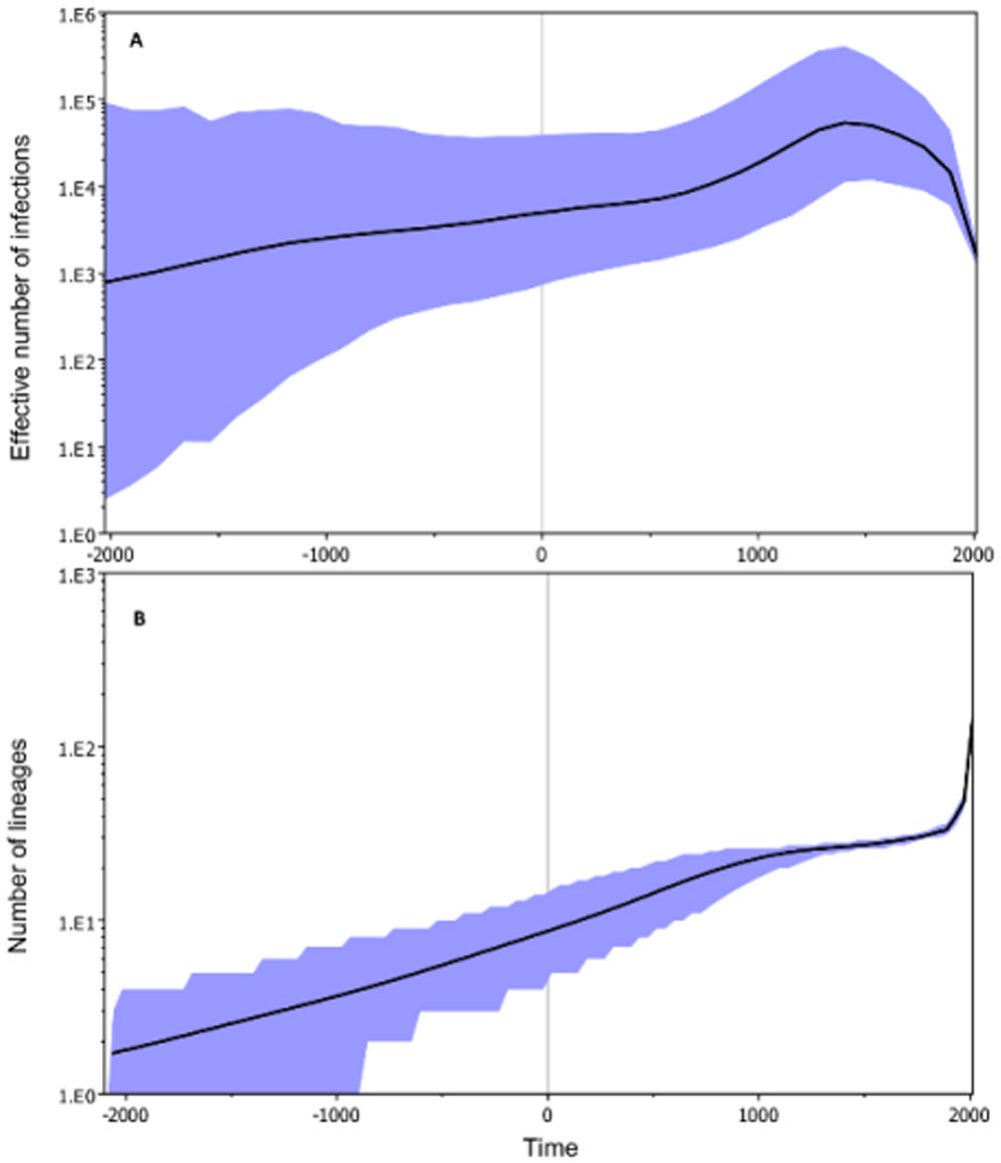
Bayesian skygrid plots (BSP), inferred from partial *L*-*gene*, for Rodentia associated *UMRV*. BSP depict viral population dynamics and the changing levels of genetic diversity (**A**) and the effective number of viral lineages (**B**) (y axis; log10 scale) over time (x axis; calendar years) for Rodentia associated *UMRV* lineages, showing the median estimate (solid line) and credibility interval (blue area). The vertical dotted line represents the upper limit of the root height, with the mean T_MRCA_ at the origin.

Analysis of the skygrid plot (Fig. 3A) showed at a global level that the effective number of Rodentia *UMRV* infections increased rapidly until around 1000 y.b.p. A further sharp increase in the number of infections occurred between 1000 and 600 y.b.p., when it reached a peak, and then epidemic diversity growth leveled-off and even decreased, though remaining at a level higher than at the start. The effective number of viral lineages depicted in Fig. 3B indicates increasing diversification of *UMRV* strands over time through three well-defined phases: phase 1, between the distant ancestor and the 11^th^ century; phase 2, between the 11^th^ century and 1900, with a linear relationship of diversification over the time and a faster rate respective to phase 1; phase 3, from about 1900 to present with nonlinear and nearly doubling in diversification in less than 100 years.

### *UMRV* evolution: selection pressures

The selection pressure analyses revealed that purifying selection is acting as the principal evolutionary force, with an abundance of negatively selected sites and there is little evidence for positive selection. Moreover, we computed different codon-specific substitution likelihood-based methods (Table 2) that accounted for discrete individual codon sites compared to *Peste*-*des*-*petits*-*ruminants virus* (PPRV), *Measles virus* (MeV), *Canine distemper virus* (CDV) and *Feline morbillivirus* (FMV) for the same *L*-*gene* region^17^. Codon-specific-analyses indicated that not one of the studied viruses showed evidence of positive selection (Table 2), except for *UMRV* with three positively selected sites inferred with the Mixed Effects Model Evolution (MEME) at a significance value of *p* < 0.1, and one positively selected site at *p* < 0.05. The latter model is the most appropriate to detect episodic diversifying selection affecting discrete codon sites. Interestingly, for the DEPS method using amino acid sequences to identify directional evolution towards residues at sites and valuable for the detection of selective sweeps, there is strong evidence that some sites are evolving under directional selection in Rodentia associated *UMRV*. This is probably not the case for morbilliviruses despite the small number of tested sequences. Indeed, no evidence of recombination was detected among *Morbilli* and *Morbilli*-*related* viruses.

**Table 2:** Summary of selection pressures acting in Rodentia *UMRV* versus Morbilliviruses.

Virus genus	Codon-based nucleic acid gene level												Protein gene level		
	No of seq (partial *L*-*gene*)	D Tajima value	SLAC^a^		FEL^a^		REL^a^		MEME^a^ (codon position)	FUBAR^b^	Integrative selection (at least one method)	BGM^c^ (codon position)	No of seq (partial *L*-*gene*)	DEPS (No residues)	FADE
			Positively/negatively selected sites												
			Pos.	Neg.	Pos.	Neg.	Pos.	Neg.							
Rodent *UMRV*	120	+4.2839	None	131	None	138	NA	NA	3 (31, 62, 85)	142	146	3 (30, 84, 94)	80	62 (19)	72
PPRV	15	+0.0925	None	12	None	24	None	71	None	22	71	None	6	None	4
MeV	17	−1.0352	None	6	None	11	None	None	None	7	11	None	10	None	4
CDV	11	−0.4934	None	6	None	19	None	None	None	14	20	None	6	None	1
FMV	6	−0.2620	None	1 (109)	None	28	None	None	None	12	28	None	5	None	None

^a^ *p* < 0.1 (no positively selected sites were detected, except for one site (62) with MEME at *p* < 0.05). ^b^Posterior probability >0.90. ^c^Posterior probability >0.95. seq: sequences.

For nucleic acid level, we used the HKY85 model, except for Rod-*UMRV* (GTR) with input neighbor-joining trees. For protein gene level, we used the JTT model. Directional Evolution Protein Sequences (DEPS) in *UMRV* (selective sweeps): 23 residues substitutions evolving under a Frequency-dependent selection, 73 residues substitutions evolving under a Convergent evolution and 18 sites evolving under a Balancing selection (Frequency-dependent selection versus Convergent evolution). Except for the D Tajima statistical test (MEGA6), all the analyses have been performed via the Datamonkey facility.

## Discussion

In order to test the robustness of our phylogenies for the *Paramyxoviridae*, at both the level of genus and species, using a portion of the *L*-*gene*, we first performed a comparative phylogenetic analysis between this fragment and the full *L*-*gene*. The results are congruent between partial and full *L*-*gene* (Supplementary Fig. S1 and Supplementary Table S1). Thus, for the *Paramyxoviridae*, we employed the partial *L*-*gene* for phylogenetic analyses; this strategy is simply logical, as for the majority of *UMRV*, complete *L*-*gene* sequences are not available. In the phylogenetic trees, some of the rodent *UMRV* are located in a basal position relative to morbilliviruses (Supplementary Fig. S1), which in turn supports, as previously suggested^12^, a possible Rodentia origin for the emergence of *Morbillivirus*.

In order to augment available information on *UMRV* worldwide, we studied for the first time, PV infection in rodents from northern Africa. The rate of PV infection in four wild rodent species (*P*. *obesus*, *M*. *shawi*, *C*. *gundi* and *R*. *rattus*) captured within natural biotopes in central and southern Tunisia was estimated at 30.6%; the highest rates occurred in *P*. *obesus* (50.0%) and *C*. *gundi* (47.5%). These rates are to our knowledge the highest reported so far in rodents^4, 5, 18^, with the exception of 68% recently reported in introduced populations of *Sciurus carolinensis* and native populations of *S*. *vulgaris* from the United Kingdom^19^.

Phylogenetic analysis shows that the RodPV-Tun sequences belong to *UMRV*, as previously reported in other small mammal species^4, 5, 18^, further emphasizing the broad geographic distribution of these viruses. Until recently, the Rodentia taxa harboring *UMRV* included four families Muridae, Cricetidae, Sciuridae and Nesomyidae^4, 5, 18, 19^. The present study adds an additional rodent family, Ctenodactylidae (*C*. *gundi*), and two additional species of murids (*P*. *obesus* and *M*. *shawi*) as potential reservoir hosts.

Phylogenetically, RodPV-Tun is divided into two major clades. The first clade contains all sequences from *P*. *obesus*, *M*. *shawi* and most from *C*. *gundi*, while the second clade includes those from *R*. *rattus* and some from *C*. *gundi*. The sedentary behavior of *P*. *obesus*, which lives in a distinct halophytic habitat^20^, may account for the specific viral strain in this species. In contrast, the other native rodent species, i.e. *C*. *gundi*, use a broader range of habitats and have greater dispersal capacity, and, hence, because of a lack of ecological isolation are infected by the viral *UMRV* shared with *Rattus*. The apparent specificity in *P*. *obesus* may also reflect a sampling bias and additional collections of *R*. *rattus* trapped in vicinity of *P*. *obesus* or a broader geographic sampling of *P*. *obesus* might clarify this issue.

At the global level, *UMRV* are phylogenetically divided into three major lineages: one restricted to the Afro-Malagasy region, including the SWIO islands, and the two others are known from different localities across the earth. The grouping of viruses originating from distant locations, such as Tunisia and the SWIO islands or Trinidad/Tobago and Australia, attests that these different strain sequences do not segregate simply on the basis of their geographical origin.

Of particular note, the phylogenetic tree retains at the global level (Fig. 1, and Supplementary Fig. S3) the same topology observed in RodPV-Tun (Supplementary Fig. S2), supporting the high diffusion capacity of these viruses to infect a wide range of rodents. Viral sequences from *C*. *gundi*, a species endemic to North Africa, clustered in two separate clades with *UMRV* sequences from *E*. *minor*, an endemic species to Madagascar. The fact that the reservoir species harboring genetically related *UMRV* are scattered in distant portions of the planet, strongly suggests the involvement of a third animal host disseminating the *UMRV*. Members of the genus *Rattus* are ideal candidates to play the role of main diffusing vector. Indeed, the sequences from *C*. *gundi* (Tunisia) and *E*. *minor* (Madagascar) closely cluster with viral sequences detected in *R*. *rattus* from these two countries; the natural range of *R*. *rattus* is southern Asia. Several infection spillover events are detected in our dataset, all involving *R*. *rattus* in different combinations: *E*. *minor*/*R*. *rattus*, *C*. *gundi*/*R*. *rattus*, *M*. *natalensis*/*R*. *rattus* and *R*. *norvegicus*/*R*. *rattus*. Similar exchanges of *UMRV* between *R*. *rattus* and other animals belonging to the orders Chiroptera and Afrotheria have already been described^5^, further stressing the central role of *R*. *rattus* in the transmission across different mammal orders and the worldwide dissemination of *UMRV*.

Our phylogeographic model (Fig. 2) supports this view and we propose that the current broad distribution of *UMRV* is directly related to the advent of global trade^21^, which resulted in the introduction of infected rats over the five continents, as has been described for other zoonoses^22–24^. Following the phylogeographic reconstruction presented herein, invasion of rodent associated *UMRV* originated some 4000 y.b.p. and indicates southern Africa as the center of origin. Some 500 years later, *UMRV* split into two major viral lineages, but with the conservation of the ancestral strain: clade B, associated with some SWIO islands and African viruses, around 2200 y.b.p. and clade A1, which diverged into strains distributed across the globe, around 3000 y.b.p.

*J* and *Mossman viruses* originating from Australia, belonging to two different clades, are closely related to German and Zambian *UMRV* and were introduced to Australia between 1400 and 1800 y.b.p., respectively. Similarly, *Nariva virus* from Trinidad/Tobago is closely related to Zambian viruses and was introduced to the Caribbean about 1600 y.b.p. *Beilong* and *Tailam viruses* from China are related to viruses occurring on SWIO islands (except Madagascar), and were introduced some 1000 y.b.p. The most recent common ancestor (T_MRCA_) of Rodentia associated *UMRV* from Germany and Tunisia dates back to 2150 and 2250 y.b.p., respectively.

Previous studies have found clear evidence in the subfamily Murinae for natural dispersal events over the last 12 million years between different portions of Africa and Asia^25^, as well as presumed anthropogenic introductions for *R*. *rattus* in prehistoric times^26^. As further evidence of the dispersal capacity of *R*. *rattus* mediated by human movements and trade, we found that dates of virus introduction differ between the SWIO islands: the T_MRCA_ indicates dates between 2100 and 1200 y.b.p. for La Réunion and Madagascar, respectively, and 150 y.b.p. for the Seychelles and Mayotte. These dates are congruent with previous estimates of when *Rattus* were introduced to SWIO islands^26–28^. The exception is La Réunion, where humans are thought to have first colonized early in the early 16^th^ century, although earlier human landing is plausible using ancient traveler descriptions^27^. The 2100 y.b.p. date is based on inference from three sequences (two from Zambian *Mus minutoides* and a single sequence from *R*. *rattus* from La Réunion). For Madagascar, our results are consistent with previous estimates that places *R*. *rattus* introduction at around 3000 before present^26, 28^. For South Africa, dates for the first introduction of *R*. *rattus* have not been precise, but some authors suggest this took place directly from India and/or the Middle East, possibly via early Arab trading activities between the 8^th^ and 9^th^ centuries^26^. Our data indicate circulation of *UMRV* in South African *Rhabdomys pumilio* dating back to around 250–500 y.b.p.

The possibility of different dates of virus introduction in the same geographic region can be explained by the occurrence of multiple introductions of *R*. *rattus*, as has been shown for Madagascar^28, 29^. Likewise, the evolutionary history of *R*. *rattus* described by Aplin *et al*.^26^ suggests that commensalism directly linked to human cultural history arose on several occasions in different geographic populations of *R*. *rattus*, and would have important implications for host-pathogen coevolution in murid rodents^21, 24^. Further, in different portions of the world two different introduced species of *Rattus* occur, often living in different ecological conditions, such as *R*. *rattus* and *R*. *norvegicus* on SWIO islands, which would suggest an additional element to multiple virus introductions.

Analysis of viral population dynamics (Fig. 3A) revealed that *UMRV* genetic diversity increased continuously until around 1000 y.b.p., followed by an exponential increase starting around the 10–11^th^ century, and reaching a peak in about the 16^th^ century. In terms of viral diversity, this scenario can be explained first by the initiation and then deeper lineage diversification, leading to a faster increase of the polymorphisms necessary to create new viral lineages from Africa (Fig. 3B). The decline in genetic diversity commencing in the 16^th^ century reflects a dramatic overall reduction of viral diversity. This phenomenon may be the result of different events associated with viral evolution: (i) an ancient drastic recombination event occurred in the evolutionary history of this group; or (ii) a founder effect with multiple host-jumps, associated with large-scale geographical expansion, and then a global population bottleneck that purged preexisting genetic diversity and was selectively determined.

In considering the first hypothesis, we cannot exclude remote infrequent recombination events^30^, but it is difficult to detect such a phenomenon, as multiple viral generations have taken place and purifying selection can obscure the ancient age of viral lineages^31^. Moreover, the presence of some breakpoints could be attributed to heterotachy, an important process in protein evolution^32^. However, these aspects are not the most suitable explanations, as recombination can shuffle mutations and purge deleterious variants, generating genotypes adapted to new environments, and still does not explain the trend of genetic diversity contraction starting sometime around the 16^th^ century. Regarding the second hypothesis, associated with the founder effect, this might provide a possible explanation, for both the increase in viral diversification through time and at different geographical points, and the boost of genetic drift with mutation accumulations, would lead to a greater loss of diversity^33^. Actually, the colonization by a few founders during periods of expansion, occurring on multiple occasions, and at a large geographic scale, can create population bottlenecks and an iterative loss of genetic diversity^34^. This aspect is in agreement with our data, specifically the positive D Tajima value estimate associated with the shift in genetic composition owing to sampling effects. Hence, *UMRV* global evolution would have been driven by genetic drift compensated by major purifying selection. This evolutionary mode was influenced by its effect on frequency distributions of trait values, and the slightly more anecdotic regimes of balancing selection (frequency-dependent selection versus convergent selection) because of multiple constraint combinations. Moreover, the weak evidence that some codons could be evolving under positive selection indicates possible adaptive fine-tuning (micro-evolution) because of cross-species transmission^8, 35^.

The 11^th^ century, the period when *UMRV* expanded in diversity, represented a dramatic turning point in human Old World history. This is the beginning of the Christian Crusades that were accompanied with considerable population displacement and mixing, large-scale expansion of international trade^36^, urbanization, and changes in land use patterns^37, 38^. The recurrent bouts of bubonic plague due to *Yersinia pestis* in which *Rattus* played the role of disseminating vector, illustrate the health consequences of maritime exchanges in the context of global cultural evolution^24, 39^. In this context, the evolutionary history of Rodentia *UMRV* seems particularly connected to the human-induced dispersal history of *R*. *rattus*. This is attested by the increase in the number of lineages with the concomitant decline in genetic diversity observed among the PV population (Fig. 3). Strikingly, in all *UMRV* studied here, our estimates of the genetic diversity for the partial *L*-*gene*, are between 1000 and 5000 and, hence, are considerably higher than those previously recorded for the structural *N* and *H* genes in MeV^40^ and PPRV^41^, and reported as being unexpectedly low. However, our results are congruent with levels of measured genetic diversity for other viruses such as HIV^42^ and hepatitis C^43^. From an epidemiological point of view, the increased number of *UMRV* lineages probably reflects *R*. *rattus* establishing epidemiological bridges between different mammal species. Since the 16^th^ century, multiple exploratory expeditions from Europe to elsewhere in the world^44^ augmented the anthropogenic dispersal of *Rattus*. On Madagascar, for example, this has also coincided with increased human ecological pressure^45^ that have facilitated *R*. *rattus* invasion and the expansion of this alien species into native forest habitats, where it entered into contact with endemic rodents of the subfamily Nesomyinae^46^. In parallel, Europe and Africa experience an intensification of agriculture and modern farming^47, 48^, which in turn allowed expansion of the geographical range of this rodent. All these elements support the role of *R*. *rattus* (and possibly, based on limited data presented herein, *R*. *norvegicus*), as a super diffusing pathogen vector at the local, regional and global scales. Further studies are needed to evaluate how other mammalian host reservoirs, such as members of the orders Chiroptera and Afrotheria, might have fueled the natural history of *UMRV* expansion and genetic evolution.

To our knowledge, this is the first evidence described to date where evolution dynamics for a zoonotic virus, originating from and hosted by wild animals, is overlaid on the population global expansion of a reservoir host tightly associated with human activities. Further investigations are warranted to unravel the specificity of *UMRV*-reservoir host interactions, the consequences of infection by these viruses in humans and animals, as well as their close phylogenetic relationships with the genus *Morbillivirus*. This in turn should help resolve how viruses evolve and predict future emergence of infections with epidemic and/or pandemic potential.

## Methods

### Ethics Statement

The research protocol was approved by the institutional review board of the Institut Pasteur de Tunis. All new animal collections and sampling, as well as associated laboratory work, were conducted in Tunisia and followed the guidelines of International Guiding Principles for Biomedical Research Involving Animals. The other sequence data used herein employed samples held by the Centre de Recherche et de Veille sur les maladies Emergentes dans l’Océan Indien (CVROI), La Réunion, France or downloaded from Genbank.

### Sampling of rodents in Tunisia

As previously described, between 2009 and 2013, 784 Tunisian wild rodents were trapped^20^ in five districts and in two different governorates: Sidi Bouzid in the humid central area and Tataouine in the arid south (Supplementary Fig. S2). Trapped rodents were transferred alive to the laboratory for physical examination and tissue sampling. Relevant parameters were recorded for each animal: species identification, sex and standard morphometric measurements. After cardiac exsanguination, heart, liver, kidney, spleen, lungs and brain samples were collected and immediately frozen in liquid nitrogen and stored at −80 °C until analysis.

Pearson Chi-Square and Fisher exact tests allowed comparison of categorical variables. STATA software version 11 was used to carry out all statistical analysis.

### Paramyxoviruses detection by RT-PCR

For each animal, total nucleic acids were extracted from approximately 1 mm^3^ of lung, kidney and spleen using the viral mini kit v2.0 and an EZ1 BioRobot (QIAGEN). Tissues were homogenized in DMEM medium by TissueLyser (QIAGEN) for 2 min at 25 Hz using 3 mm tungsten beads. cDNA was generated through reverse transcription (Promega cDNA kit). Semi-nested PCR targeting a partial sequence of the *L*-*gene* polymerase locus (∼490 bp) of *Paramyxovirinae* subfamilies was carried out using previously described conditions^5, 49^. RT-PCR products were purified using the QIAquick PCR purification kit (QIAGEN), cloned into the pGEM-T Easy vector (Promega), according to the manufacturer’s instructions and sequenced using chain-termination (Sanger) sequencing (Big Dye Sequencing kit, ABI) on both strands by a commercial service (Genoscreen).

### Origin of viruses and dataset construction

Phylogenetic and phylogeographic analyses were conducted using the 42 partial *L*-*gene UMRV* sequences from rodents caught in Tunisia (three sequences where eliminated due to reduced number of base pairs) into a panel of 145 partial *UMRV* sequences infecting rodents from 11 countries, recovered from public databases spanning the period from 1962 to 2013. Only sequences with well-documented collection dates and location origin were employed.

Four data sets were used. The first was to compare phylogenetic analysis of the partial *L*-*gene* and the full *L*-*gene* among *Paramyxoviridae* (Supplementary Data S2). The sequences were extracted in a manner to create parallel data sets with identical origins. These two subsets of operational taxonomic units (OTUs) were defined using Mothur^50^, and based on a 90% genetic distance cutoffs, generated 179 representative sequences. The second set was recovered from GenBank, focusing on *Measles virus* (MeV) (Supplementary Table S3) and was used to estimate the evolutionary rate of the *L*-*gene* polymerase locus. The third set (Supplementary Data S3) was representative of most recognized PV genera and allowed classification of Tunisian sequences among the *Paramyxoviridae*. The last set (Supplementary Data S4) was devoted to the phylogenetic and the phylogeographic analyses of the Rodentia associated *UMRV*.

### Phylogenetic analysis: maximum-likelihood and Bayesian inference analysis

Partial *L*-*gene* sequences of PV infecting rodents from Tunisia were compared to published sequences in GenBank (National Center for Biotechnology Information, Bethesda, USA) online (www.ncbi.nih.gov) using BLASTn and BLASTx. Raw sequences were then cleaned for primer sequences, edited, assembled and compared using Geneious^®^Pro 7.1.8^51^. The multiple alignments of partial *L*-*gene* sequences were performed with the MAAFT 7.0.17 algorithm^52^.

A GTR Gamma + I nucleotide substitution model was determined to best fit the data using Akaike Information Criterion in MEGA6^53^, and in ModelTest via the integrated Geneious Paup tool^54^. The same best-fit substitution model was used for all phylogenetic analyses. Phylogenetic relationships among PV strains based on partial *L*-*gene* sequences were analyzed using both Bayesian inference and maximum likelihood (ML) methodologies. ML trees were estimated, with 1000 rapid bootstraps, employing RAxML 7.2.8 Geneious plugin, under the best-scoring ML tree parameter, and using MEGA6^53^. Bayesian phylogenetic trees were supported by the topology of the ML trees. Bayesian inference analysis was performed with the MrBayes 3.2.2 plugin included in Geneious software^55^, with constrained and unconstrained branch lengths, four Metropolis-coupled chains for 10 million Markov Chain Monte Carlo (MCMC) generations, sampling of the Markov chain every 200 generations and discarding the first 25% as burn-in. Tree constructions (Supplementary Fig. S1) were performed using 50 million and 6 millions iterations, respectively, for the partial *L*-*gene* and the full *L*-*gene*, in MrBayes with the GTR+ G+ I evolutionary model and a 10% burn-in rooted with the *Sunshine* paramyxovirus sequence (JN192445). The sequences of bat-rabies virus (JQ595353) in Supplementary Fig. S2 and rodents PV (RodPVs) (HQ660195 and AB844426, 2 *Respirovirus* sequences) in Fig. 1 and Supplementary Fig. S3 were chosen as outgroup. Trees were visualized by FigTree 1.4.2 (www.tree.bio.ed.ac.uk/software/figtree/).

The applicability of a molecular clock to the data set was evaluated with a log-likelihood-ratio test using the ML scores, with and without a molecular clock enforced. The null hypothesis of equal evolutionary rate throughout the trees was not rejected at a 5% significance level when assessed using MEGA6^53^. Therefore, all subsequent phylogenetic analyses were performed using a strict-molecular clock. For the Bayesian tree reconstructions, no difference was observed between strict- and relaxed-molecular clocks. However, for the phylogenetic analysis performed with Beast package 1.8.2^56^, we used a relaxed-clock Bayesian MCMC method, as previous studies have shown that it allowed a better data fit than a strict clock^56–58^.

### Bayesian evolutionary analyses of spatio-temporal *UMRV* dynamics

To explore the evolutionary relationships and times to the most recent common ancestors (T_MRCA_) among *UMRV* lineages circulating at a global level, we reconstructed phylogenetic history using Bayesian MCMC analysis implemented in the Beast package. *UMRV* sequences were annotated to one of 11 locations based on country of origin (Australia, China, Germany, Madagascar, Mayotte, La Réunion, Seychelles, South Africa, Trinidad/Tobago, Tunisia and Zambia), and used as a discrete trait during MCMC analysis. We employed a GTR+ G+ I model of nucleotide substitution with a normally distributed rate variation among sites (3.48 × 10^−4^, sd 1.69 × 10^−5^, values obtained from the MeV data set analysis (Supplementary Text S2 and Supplementary Table S3) and a relaxed (uncorrelated log-normal) molecular clock model. First, we specified a Bayesian skyline population coalescent model (10 piece-wise constant groups)^57^, then, we investigated, with the same prior distribution, a non-parametric skygrid plot model^59^. All chains convergence was assessed using Tracer, with statistical uncertainty reflected in values of the 95% highest posterior density, removing at least 10% of the chains as burn-in. The probable locations of each ancestral node and evolutionary time past were summarized using an annotated Maximum Clade Credibility (MCC) phylogenetic tree.

Lemey *et al*.^60^ have implemented a continuous-time Markov chain (CTMC) over discrete sampling locations in the Beast package, making it possible to model spatial diffusion on a time-scaled phylogenetic tree^60^. To determine the different lineage migration patterns, inferred with a Bayesian stochastic search variable selection scheme (BSSVS), allowing the switch rates in the CTMC to be zero with some prior probability, we used either a CTMC symmetric (reversible: bidirectional between locations) or asymmetric (non-reversible: unidirectional route) substitution models for discrete geographic traits. Coalescent models were compared using a modified Akaike information criterion (AICM) in Tracer^61^. The BSSVS procedure computes the most parsimonious possible rate values, by assigning a Bayes factor (BF) significance test between transmission routes and explaining the different forms of diffusion. The result was calculated and visualized by Spread^62^; a BF cutoff greater than three was taken as significant support, as previously reported^60, 63^.

### Selection pressure

First, we used a series of tests provided by MEGA6^53^. We conducted Tajima’s test of neutrality, a statistical test to compare the number of segregating sites per site with the nucleotide diversity, specifically to determine if sequences are randomly evolving (neutrally), as compared to those driven by non-stochastic processes (directional selection, balancing selection, demographic expansion or contraction, etc.). A Tajima’s D = 0, indicates that the population dynamic evolves without evidence of selection. When D < 0, it means that population size is not at equilibrium, but expanding, due to an over representation of infrequent polymorphisms, typical after a bottleneck or a selective sweep and/or purifying selection. In cases when D > 0, this represents a decrease in population size and/or balancing selection due to an under representation of both low and high frequency polymorphisms. Based on the numbers of Synonymous (*d*_*S*_) and Nonsynonymous (*d*_*N*_) substitutions between sequences, a codon-based Fisher’s exact test was conducted, and also an Estimate Selection for each codon (HyPhy). The latter computes the selection robustness, whether negative or positive, acting on each corresponding codon and provides statistical estimates. Considering the rather large size of our dataset, we made also a Z-Test to test the null hypothesis that H_0_: *d*_*N*_ = *d*_*S*_. The level of significance at which H_0_ is rejected depends on the respective alternative hypotheses (HA): (a), (b), or (c) with (a) *d*_*N*_ = *d*_*S*_ (test of neutrality), (b) *d*_*N*_ > *d*_*S*_ (positive selection), (c) *d*_*N*_ < *d*_*S*_ (purifying selection). Selection pressures were also measured using SLAC/REL/FEL maximum likelihood methods, MEME, FUBAR, BGM, and DEPS/FADE via the Datamonkey facility^17^. The recombination detection program (RDP) implemented in the RDP 4.46 software package^64^ was also used.

### Data accessibility

Forty-five viral sequences of *UMRV* from rodents captured in Tunisia were generated in the present study and were deposited in GenBank with the accessions numbers: KJ408384-KJ408428.

## Electronic supplementary material

Supplementary Information

Supplementary Data S1

Supplementary Data S2

Supplementary Data S3

Supplementary Data S4
