# Supplementary material for: Insight into the global evolution of Rodentia associated Morbilli-related paramyxoviruses
Source: Sci Rep. 2017 May 16;7:1974. doi: 10.1038/s41598-017-02206-0 (PMC5434063; doi:10.1038/s41598-017-02206-0)
Supplement: Supplementary file 1 — Supplementary Information [file 41598_2017_2206_MOESM1_ESM.pdf]

Supplementary Information

Insight into the global evolution of Rodentia associated *Morbilli-related* paramyxoviruses

Wissem Ghawar[1,2,3,4,¶,*], Hervé Pascalis[1,5,¶,*], Jihéne Bettaieb[2,3,4], Julien Mélade[1,5], Adel Gharbi[2,3,4], Mohamed Ali Snoussi[2,3,4], Dhafer Laouini[3,4], Steven M. Goodman[6,7], Afif Ben Salah[2,3,4], Koussay Dellagi[1,3,5].

[1]Centre de Recherche et de Veille sur les maladies émergentes dans l'Océan Indien (CRVOI), Plateforme de Recherche CYROI, Sainte Clotilde, La Réunion, France.

[2]Laboratory of Medical Epidemiology, Institut Pasteur de Tunis (IPT), Tunis-Belvédère, Tunis, Tunisia.

[3]Laboratory of Transmission, Control and Immunobiology of Infections (LTCII), LR11IPT02, Institut Pasteur de Tunis (IPT), Tunis-Belvédère, Tunis, Tunisia.

[4]Université Tunis El Manar, Tunis, Tunisia.

[5]Université de La Réunion, UMR PIMIT "Processus Infectieux en Milieu Insulaire Tropical", INSERM U1187, CNRS 9192, IRD 249, Plateforme de Recherche CYROI, Saint Denis, La Réunion, France.

[6]Field Museum of Natural History, 1400 S. Lake Shore Dr, Chicago, IL 60605-2496, USA.

[7]Association Vahatra, BP 3972, Antananarivo 101, Madagascar.

[*]Authors for correspondence: Wissem Ghawar, e-mail: ghawarwissemmed@yahoo.fr and Hervé Pascalis, e-mail: herve.pascalis@ird.fr.

[¶]Wissem Ghawar and Hervé Pascalis contributed equally to this work.

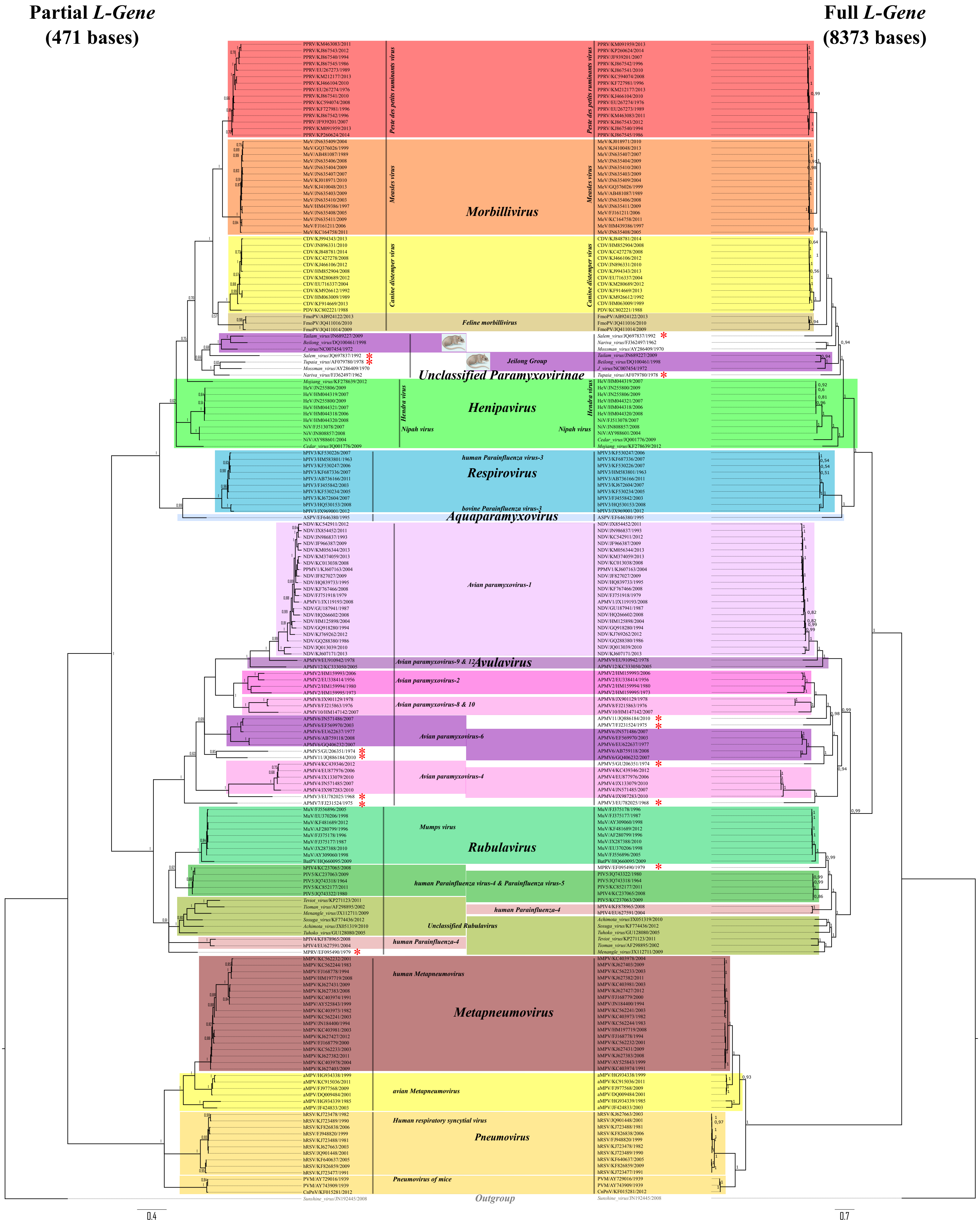

Partial L-Gene
(471 bases)
Full L-Gene
(8373 bases)
Peste des petits ruminants virus
Measles virus
Morbillivirus
Canine distemper virus
Feline morbillivirus
Jeilong Group
Unclassified Paramyxovirinae
Hendra virus
Henipavirus
Nipah virus
human Parainfluenza virus-3
Respirovirus
bovine Parainfluenza virus-3
Aquaparamyxovirus
Avian paramyxovirus-1
Avian paramyxovirus-9 & 12
Avulavirus
Avian paramyxovirus-2
Avian paramyxovirus-8 & 10
Avian paramyxovirus-6
Avian paramyxovirus-4
Mumps virus
Rubulavirus
human Parainfluenza virus-4 & Parainfluenza virus-5
human Parainfluenza-4
Unclassified Rubulavirus
human Metapneumovirus
Metapneumovirus
avian Metapneumovirus
Human respiratory syncytial virus
Pneumovirus
Pneumovirus of mice
Outgroup
0.4
0.7

Supplementary Figure S1: Comparative Bayesian phylogeny of the partial *L-gene* and the full *L-gene* for the *Paramyxoviridae* family. Similarity between trees at the levels of viral genera and species are depicted with color frames. Observed inconsistencies between the trees are identified with a red asterisk. Node values indicate Bayesian posterior probabilities. Virus designations are as follows: Virus name/accession number/Host/collection year. Abbreviations are detailed in Supplementary Data 2. The *Sunshine virus* was used as outgroup.

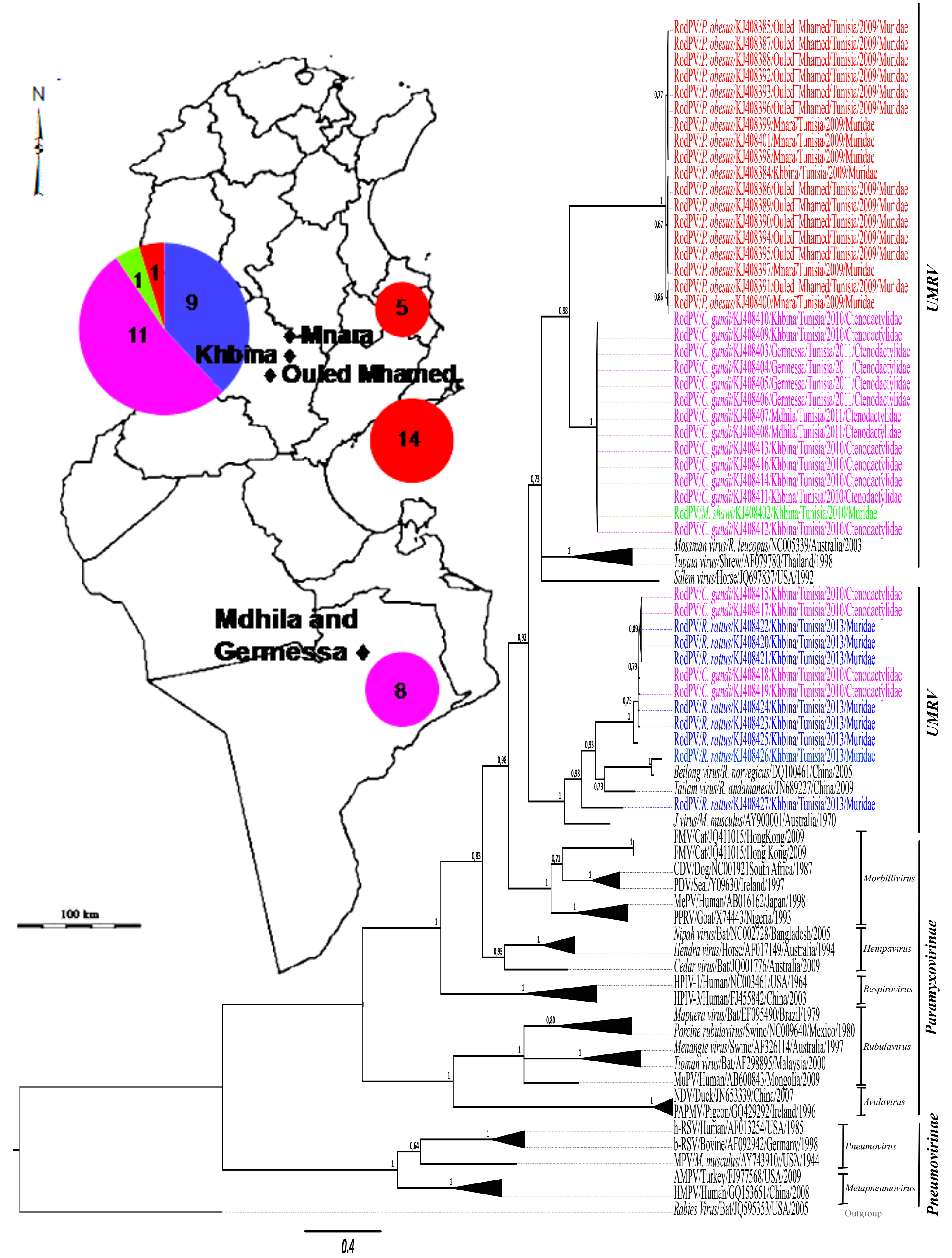
N
100 km
1
1
9
11
5
Mnara
Khbina
Ouled Mhamed
14
Mdhila and
Germessa
8
RodPV/P. obesus/KJ408385/Ouled_Mhamed/Tunisia/2009/Muridae
RodPV/P. obesus/KJ408387/Ouled_Mhamed/Tunisia/2009/Muridae
RodPV/P. obesus/KJ408388/Ouled_Mhamed/Tunisia/2009/Muridae
RodPV/P. obesus/KJ408392/Ouled_Mhamed/Tunisia/2009/Muridae
RodPV/P. obesus/KJ408393/Ouled_Mhamed/Tunisia/2009/Muridae
RodPV/P. obesus/KJ408396/Ouled_Mhamed/Tunisia/2009/Muridae
RodPV/P. obesus/KJ408399/Mnara/Tunisia/2009/Muridae
RodPV/P. obesus/KJ408401/Mnara/Tunisia/2009/Muridae
RodPV/P. obesus/KJ408398/Mnara/Tunisia/2009/Muridae
RodPV/P. obesus/KJ408384/Khbina/Tunisia/2009/Muridae
RodPV/P. obesus/KJ408386/Ouled_Mhamed/Tunisia/2009/Muridae
RodPV/P. obesus/KJ408389/Ouled_Mhamed/Tunisia/2009/Muridae
RodPV/P. obesus/KJ408390/Ouled_Mhamed/Tunisia/2009/Muridae
RodPV/P. obesus/KJ408394/Ouled_Mhamed/Tunisia/2009/Muridae
RodPV/P. obesus/KJ408395/Ouled_Mhamed/Tunisia/2009/Muridae
RodPV/P. obesus/KJ408397/Mnara/Tunisia/2009/Muridae
RodPV/P. obesus/KJ408391/Ouled_Mhamed/Tunisia/2009/Muridae
RodPV/P. obesus/KJ408400/Mnara/Tunisia/2009/Muridae
RodPV/C. gundi/KJ408410/Khbina/Tunisia/2010/Ctenodactylidae
RodPV/C. gundi/KJ408409/Khbina/Tunisia/2010/Ctenodactylidae
RodPV/C. gundi/KJ408403/Germessa/Tunisia/2011/Ctenodactylidae
RodPV/C. gundi/KJ408404/Germessa/Tunisia/2011/Ctenodactylidae
RodPV/C. gundi/KJ408405/Germessa/Tunisia/2011/Ctenodactylidae
RodPV/C. gundi/KJ408406/Germessa/Tunisia/2011/Ctenodactylidae
RodPV/C. gundi/KJ408407/Mdhila/Tunisia/2011/Ctenodactylidae
RodPV/C. gundi/KJ408408/Mdhila/Tunisia/2011/Ctenodactylidae
RodPV/C. gundi/KJ408413/Khbina/Tunisia/2010/Ctenodactylidae
RodPV/C. gundi/KJ408416/Khbina/Tunisia/2010/Ctenodactylidae
RodPV/C. gundi/KJ408414/Khbina/Tunisia/2010/Ctenodactylidae
RodPV/C. gundi/KJ408411/Khbina/Tunisia/2010/Ctenodactylidae
RodPV/M. shawi/KJ408402/Khbina/Tunisia/2010/Muridae
RodPV/C. gundi/KJ408412/Khbina/Tunisia/2010/Ctenodactylidae
Mossman virus/R. leucopus/NC005339/Australia/2003
Tupaia virus/Shrew/AF079780/Thailand/1998
Salem virus/Horse/JQ697837/USA/1992
UMRV
RodPV/C. gundi/KJ408415/Khbina/Tunisia/2010/Ctenodactylidae
RodPV/C. gundi/KJ408417/Khbina/Tunisia/2010/Ctenodactylidae
RodPV/R. rattus/KJ408422/Khbina/Tunisia/2013/Muridae
RodPV/R. rattus/KJ408420/Khbina/Tunisia/2013/Muridae
RodPV/R. rattus/KJ408421/Khbina/Tunisia/2013/Muridae
RodPV/C. gundi/KJ408418/Khbina/Tunisia/2010/Ctenodactylidae
RodPV/C. gundi/KJ408419/Khbina/Tunisia/2010/Ctenodactylidae
RodPV/R. rattus/KJ408424/Khbina/Tunisia/2013/Muridae
RodPV/R. rattus/KJ408423/Khbina/Tunisia/2013/Muridae
RodPV/R. rattus/KJ408425/Khbina/Tunisia/2013/Muridae
RodPV/R. rattus/KJ408426/Khbina/Tunisia/2013/Muridae
Beilong virus/R. norvegicus/DQ100461/China/2005
Tailam virus/R. andamanesis/JN689227/China/2009
RodPV/R. rattus/KJ408427/Khbina/Tunisia/2013/Muridae
J virus/M. musculus/AY900001/Australia/1970
UMRV
FMV/Cat/JQ411015/HongKong/2009
FMV/Cat/JQ411015/Hong Kong/2009
CDV/Dog/NC001921South Africa/1987
PDV/Seal/Y09630/Ireland/1997
MePV/Human/AB016162/Japan/1998
PPRV/Goat/X74443/Nigeria/1993
Morbillivirus
Nipah virus/Bat/NC002728/Bangladesh/2005
Hendra virus/Horse/AF017149/Australia/1994
Cedar virus/Bat/JQ001776/Australia/2009
Henipavirus
HPIV-1/Human/NC003461/USA/1964
HPIV-3/Human/FJ455842/China/2003
Respirovirus
Mapuera virus/Bat/EF095490/Brazil/1979
Porcine rubulavirus/Swine/NC009640/Mexico/1980
Menangle virus/Swine/AF326114/Australia/1997
Tioman virus/Bat/AF298895/Malaysia/2000
MuPV/Human/AB600843/Mongolia/2009
Rubulavirus
NDV/Duck/JN653339/China/2007
PAPMV/Pigeon/GQ429292/Ireland/1996
Avulavirus
Paramyxovirinae
h-RSV/Human/AF013254/USA/1985
b-RSV/Bovine/AF092942/Germany/1998
MPV/M. musculus/AY743910//USA/1944
Pneumovirus
AMPV/Turkey/FJ977568/USA/2009
HMPV/Human/GQ153651/China/2008
Metapneumovirus
Pneumovirinae
Rabies Virus/Bat/JQ595353/USA/2005
Outgroup
0.4

Supplementary Figure S2: Maximum likelihood phylogeny of the partial *L-gene* of the *Paramyxoviridae* family including the Tunisian paramyxoviruses. PV sequences are distributed according to the geographic location of sampling sites and are colored according to the host species: *Psammomys obesus*, *Meriones shawi*, *Rattus rattus* and *Ctenodactylus gundi* colored in red, green, blue and purple; respectively. Values at node points indicate Bayesian posterior probabilities. Virus designations were as follow: Virus name or virus affiliation/typical host/accession number/origin/collection year/host Family. Abbreviations used are detailed in Supplementary Data 3. *Rabies virus* (JQ595353) was used as an outgroup. Map was generated using ArcGIS software (version 10.1; https://www.arcgis.com/features/).

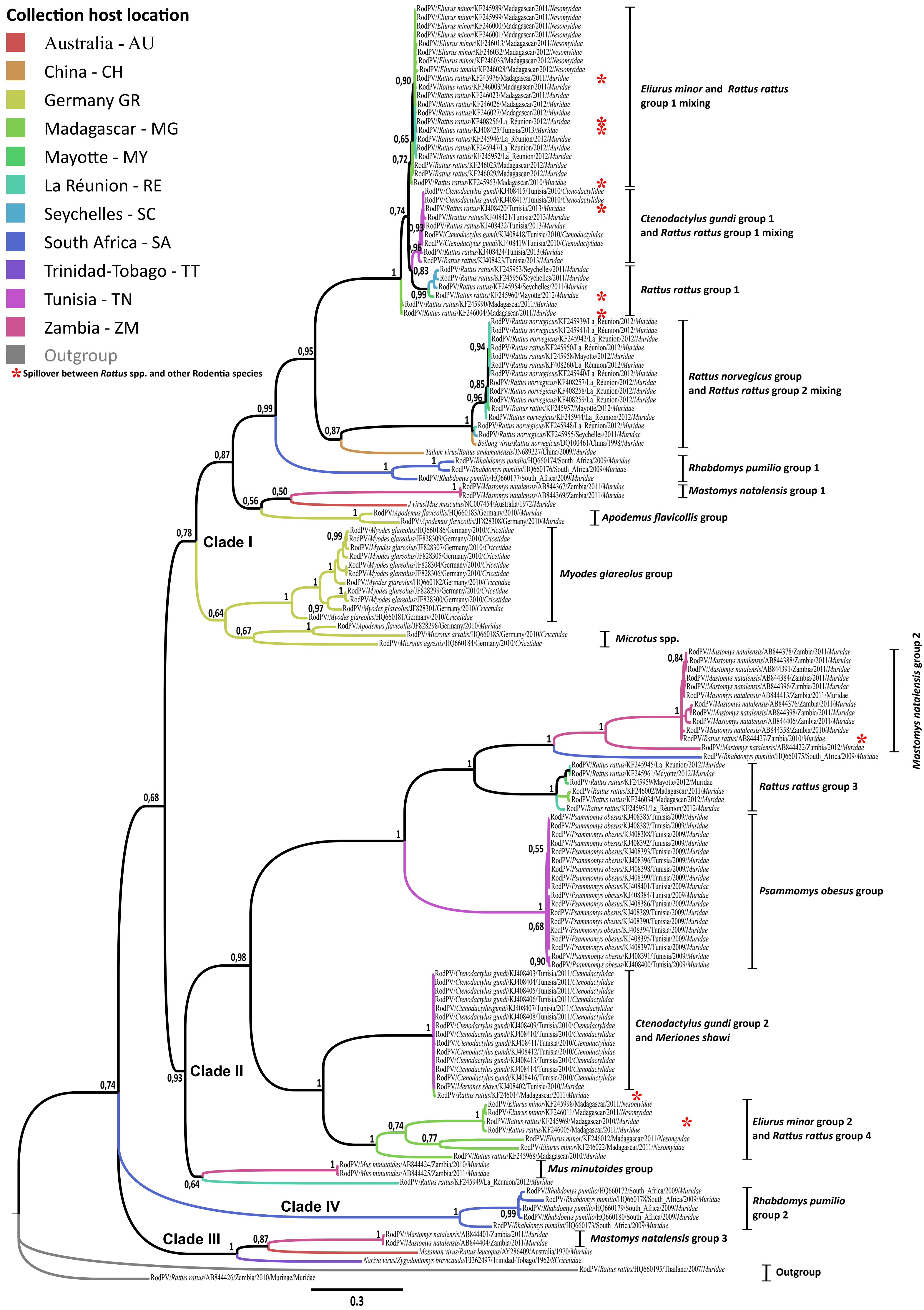

Collection host location
Australia - AU
China - CH
Germany GR
Madagascar - MG
Mayotte - MY
La Réunion - RE
Seychelles - SC
South Africa - SA
Trinidad-Tobago - TT
Tunisia - TN
Zambia - ZM
Outgroup
* Spillover between Rattus spp. and other Rodentia species
RodPV/Eliurus minor/KF245989/Madagascar/2011/Nesomyidae
RodPV/Eliurus minor/KF245999/Madagascar/2011/Nesomyidae
RodPV/Eliurus minor/KF246000/Madagascar/2011/Nesomyidae
RodPV/Eliurus minor/KF246001/Madagascar/2011/Nesomyidae
RodPV/Eliurus minor/KF246013/Madagascar/2011/Nesomyidae
RodPV/Eliurus minor/KF246032/Madagascar/2012/Nesomyidae
RodPV/Eliurus minor/KF246033/Madagascar/2012/Nesomyidae
RodPV/Eliurus tanala/KF246028/Madagascar/2012/Nesomyidae
0,90
RodPV/Rattus rattus/KF245976/Madagascar/2011/Muridae
RodPV/Rattus rattus/KF246003/Madagascar/2011/Muridae
RodPV/Rattus rattus/KF246023/Madagascar/2011/Muridae
RodPV/Rattus rattus/KF246026/Madagascar/2012/Muridae
RodPV/Rattus rattus/KF246027/Madagascar/2012/Muridae
RodPV/Rattus rattus/KF408256/La_Réunion/2012/Muridae
RodPV/Rattus rattus/KJ408425/Tunisia/2013/Muridae
0,65
RodPV/Rattus rattus/KF245946/La_Réunion/2012/Muridae
RodPV/Rattus rattus/KF245947/La_Réunion/2012/Muridae
0,72
RodPV/Rattus rattus/KF245952/La_Réunion/2012/Muridae
RodPV/Rattus rattus/KF246025/Madagascar/2012/Muridae
RodPV/Rattus rattus/KF246029/Madagascar/2012/Muridae
RodPV/Rattus rattus/KF245963/Madagascar/2010/Muridae
Eliurus minor and Rattus rattus group 1 mixing
RodPV/Ctenodactylus gundi/KJ408415/Tunisia/2010/Ctenodactylidae
RodPV/Ctenodactylus gundi/KJ408417/Tunisia/2010/Ctenodactylidae
0,74
RodPV/Rattus rattus/KJ408420/Tunisia/2013/Muridae
RodPV/Rrattus rattus/KJ408421/Tunisia/2013/Muridae
0,93
RodPV/Rattus rattus/KJ408422/Tunisia/2013/Muridae
RodPV/Ctenodactylus gundi/KJ408418/Tunisia/2010/Ctenodactylidae
RodPV/Ctenodactylus gundi/KJ408419/Tunisia/2010/Ctenodactylidae
0,96
RodPV/Rattus rattus/KJ408424/Tunisia/2013/Muridae
RodPV/Rattus rattus/KJ408423/Tunisia/2013/Muridae
Ctenodactylus gundi group 1 and Rattus rattus group 1 mixing
1
0,83
RodPV/Rattus rattus/KF245953/Seychelles/2011/Muridae
RodPV/Rattus rattus/KF245956/Seychelles/2011/Muridae
RodPV/Rattus rattus/KF245954/Seychelles/2011/Muridae
0,99
RodPV/Rattus rattus/KF245960/Mayotte/2012/Muridae
RodPV/Rattus rattus/KF245990/Madagascar/2011/Muridae
RodPV/Rattus rattus/KF246004/Madagascar/2011/Muridae
Rattus rattus group 1
0,95
RodPV/Rattus norvegicus/KF245939/La_Réunion/2012/Muridae
RodPV/Rattus norvegicus/KF245941/La_Réunion/2012/Muridae
RodPV/Rattus norvegicus/KF245942/La_Réunion/2012/Muridae
0,94
RodPV/Rattus rattus/KF245950/La_Réunion/2012/Muridae
RodPV/Rattus rattus/KF245958/Mayotte/2012/Muridae
RodPV/Rattus rattus/KF408260/La_Réunion/2012/Muridae
RodPV/Rattus norvegicus/KF245940/La_Réunion/2012/Muridae
0,85
RodPV/Rattus norvegicus/KF408257/La_Réunion/2012/Muridae
RodPV/Rattus norvegicus/KF408258/La_Réunion/2012/Muridae
0,96
RodPV/Rattus norvegicus/KF408259/La_Réunion/2012/Muridae
RodPV/Rattus rattus/KF245957/Mayotte/2012/Muridae
RodPV/Rattus norvegicus/KF245944/La_Réunion/2012/Muridae
1
0,87
RodPV/Rattus norvegicus/KF245948/La_Réunion/2012/Muridae
RodPV/Rattus norvegicus/KF245955/Seychelles/2011/Muridae
Beilong virus/Rattus norvegicus/DQ100461/China/1998/Muridae
Tailam virus/Rattus andamanensis/JN689227/China/2009/Muridae
Rattus norvegicus group and Rattus rattus group 2 mixing
0,99
1
RodPV/Rhabdomys pumilio/HQ660174/South_Africa/2009/Muridae
1
RodPV/Rhabdomys pumilio/HQ660176/South_Africa/2009/Muridae
RodPV/Rhabdomys pumilio/HQ660177/South_Africa/2009/Muridae
Rhabdomys pumilio group 1
0,87
0,50
1
RodPV/Mastomys natalensis/AB844367/Zambia/2011/Muridae
RodPV/Mastomys natalensis/AB844369/Zambia/2011/Muridae
Mastomys natalensis group 1
0,56
J virus/Mus musculus/NC007454/Australia/1972/Muridae
1
RodPV/Apodemus flavicollis/HQ660183/Germany/2010//Muridae
RodPV/Apodemus flavicollis/JF828308/Germany/2010/Muridae
Apodemus flavicollis group
0,78
Clade I
RodPV/Myodes glareolus/HQ660186/Germany/2010/Cricetidae
0,99
RodPV/Myodes glareolus/JF828309/Germany/2010/Cricetidae
RodPV/Myodes glareolus/JF828307/Germany/2010/Cricetidae
RodPV/Myodes glareolus/JF828305/Germany/2010/Cricetidae
1
RodPV/Myodes glareolus/JF828304/Germany/2010/Cricetidae
RodPV/Myodes glareolus/JF828306/Germany/2010/Cricetidae
1
RodPV/Myodes glareolus/HQ660182/Germany/2010/Cricetidae
1
RodPV/Myodes glareolus/JF828299/Germany/2010/Cricetidae
1
RodPV/Myodes glareolus/JF828300/Germany/2010/Cricetidae
0,97
RodPV/Myodes glareolus/JF828301/Germany/2010/Cricetidae
0,64
RodPV/Myodes glareolus/HQ660181/Germany/2010/Cricetidae
Myodes glareolus group
1
RodPV/Apodemus flavicollis/JF828298/Germany/2010/Muridae
0,67
RodPV/Microtus arvalis/HQ660185/Germany/2010/Cricetidae
RodPV/Microtus agrestis/HQ660184/Germany/2010/Cricetidae
Microtus spp.
RodPV/Mastomys natalensis/AB844378/Zambia/2011/Muridae
0,84
RodPV/Mastomys natalensis/AB844388/Zambia/2011/Muridae
RodPV/Mastomys natalensis/AB844391/Zambia/2011/Muridae
RodPV/Mastomys natalensis/AB844384/Zambia/2011/Muridae
RodPV/Mastomys natalensis/AB844396/Zambia/2011/Muridae
RodPV/Mastomys natalensis/AB844413/Zambia/2011/Muridae
RodPV/Mastomys natalensis/AB844376/Zambia/2011/Muridae
1
RodPV/Mastomys natalensis/AB844398/Zambia/2011/Muridae
RodPV/Mastomys natalensis/AB844406/Zambia/2011/Muridae
1
RodPV/Mastomys natalensis/AB844358/Zambia/2010/Muridae
RodPV/Rattus rattus/AB844427/Zambia/2010/Muridae
RodPV/Mastomys natalensis/AB844422/Zambia/2012/Muridae
1
RodPV/Rhabdomys pumilio/HQ660175/South_Africa/2009/Muridae
Mastomys natalensis group 2
0,68
1
RodPV/Rattus rattus/KF245945/La_Réunion/2012/Muridae
RodPV/Rattus rattus/KF245961/Mayotte/2012/Muridae
RodPV/Rattus rattus/KF245959/Mayotte/2012/Muridae
1
RodPV/Rattus rattus/KF246002/Madagascar/2011/Muridae
RodPV/Rattus rattus/KF246034/Madagascar/2012/Muridae
RodPV/Rattus rattus/KF245951/La_Réunion/2012/Muridae
Rattus rattus group 3
RodPV/Psammomys obesus/KJ408385/Tunisia/2009/Muridae
RodPV/Psammomys obesus/KJ408387/Tunisia/2009/Muridae
RodPV/Psammomys obesus/KJ408388/Tunisia/2009/Muridae
RodPV/Psammomys obesus/KJ408392/Tunisia/2009/Muridae
0,55
RodPV/Psammomys obesus/KJ408393/Tunisia/2009/Muridae
RodPV/Psammomys obesus/KJ408396/Tunisia/2009/Muridae
RodPV/Psammomys obesus/KJ408398/Tunisia/2009/Muridae
RodPV/Psammomys obesus/KJ408399/Tunisia/2009/Muridae
RodPV/Psammomys obesus/KJ408401/Tunisia/2009/Muridae
RodPV/Psammomys obesus/KJ408384/Tunisia/2009/Muridae
1
RodPV/Psammomys obesus/KJ408386/Tunisia/2009/Muridae
1
RodPV/Psammomys obesus/KJ408389/Tunisia/2009/Muridae
RodPV/Psammomys obesus/KJ408390/Tunisia/2009/Muridae
0,68
RodPV/Psammomys obesus/KJ408394/Tunisia/2009/Muridae
RodPV/Psammomys obesus/KJ408395/Tunisia/2009/Muridae
RodPV/Psammomys obesus/KJ408397/Tunisia/2009/Muridae
RodPV/Psammomys obesus/KJ408391/Tunisia/2009/Muridae
0,90
RodPV/Psammomys obesus/KJ408400/Tunisia/2009/Muridae
Psammomys obesus group
0,98
RodPV/Ctenodactylus gundi/KJ408403/Tunisia/2011/Ctenodactylidae
RodPV/Ctenodactylus gundi/KJ408404/Tunisia/2011/Ctenodactylidae
RodPV/Ctenodactylus gundi/KJ408405/Tunisia/2011/Ctenodactylidae
RodPV/Ctenodactylus gundi/KJ408406/Tunisia/2011/Ctenodactylidae
RodPV/Ctenodactylusgundi/KJ408407/Tunisia/2011/Ctenodactylidae
RodPV/Ctenodactylus gundi/KJ408408/Tunisia/2011/Ctenodactylidae
1
RodPV/Ctenodactylus gundi/KJ408409/Tunisia/2010/Ctenodactylidae
RodPV/Ctenodactylus gundi/KJ408410/Tunisia/2010/Ctenodactylidae
RodPV/Ctenodactylus gundi/KJ408411/Tunisia/2010/Ctenodactylidae
RodPV/Ctenodactylus gundi/KJ408412/Tunisia/2010/Ctenodactylidae
RodPV/Ctenodactylus gundi/KJ408413/Tunisia/2010/Ctenodactylidae
RodPV/Ctenodactylus gundi/KJ408414/Tunisia/2010/Ctenodactylidae
RodPV/Ctenodactylus gundi/KJ408416/Tunisia/2010/Ctenodactylidae
RodPV/Meriones shawi/KJ408402/Tunisia/2010/Muridae
RodPV/Rattus rattus/KF246014/Madagascar/2011/Muridae
Ctenodactylus gundi group 2 and Meriones shawi
0,93
Clade II
1
RodPV/Eliurus minor/KF245998/Madagascar/2011/Nesomyidae
1
RodPV/Eliurus minor/KF246011/Madagascar/2011/Nesomyidae
RodPV/Rattus rattus/KF245969/Madagascar/2010/Muridae
0,74
RodPV/Rattus rattus/KF246005/Madagascar/2011/Muridae
1
0,77
RodPV/Eliurus minor/KF246012/Madagascar/2011/Nesomyidae
RodPV/Eliurus minor/KF246022/Madagascar/2011/Nesomyidae
RodPV/Rattus rattus/KF245968/Madagascar/2010/Muridae
Eliurus minor group 2 and Rattus rattus group 4
1
RodPV/Mus minutoides/AB844424/Zambia/2010/Muridae
RodPV/Mus minutoides/AB844425/Zambia/2011/Muridae
Mus minutoides group
0,64
RodPV/Rattus rattus/KF245949/La_Réunion/2012/Muridae
0,74
RodPV/Rhabdomys pumilio/HQ660172/South_Africa/2009/Muridae
RodPV/Rhabdomys pumilio/HQ660178/South_Africa/2009/Muridae
Clade IV
1
RodPV/Rhabdomys pumilio/HQ660179/South_Africa/2009/Muridae
0,99
RodPV/Rhabdomys pumilio/HQ660180/South_Africa/2009/Muridae
RodPV/Rhabdomys pumilio/HQ660173/South_Africa/2009/Muridae
Rhabdomys pumilio group 2
Clade III
1
0,87
1
RodPV/Mastomys natalensis/AB844401/Zambia/2011/Muridae
RodPV/Mastomys natalensis/AB844404/Zambia/2011/Muridae
Mastomys natalensis group 3
Mossman virus/Rattus leucopus/AY286409/Australia/1970/Muridae
Nariva virus/Zygodontomys brevicauda/FJ362497/Trinidad-Tobago/1962/SCricetidae
RodPV/Rattus rattus/HQ660195/Thailand/2007/Muridae
Outgroup
RodPV/Rattus rattus/AB844426/Zambia/2010/Murinae/Muridae
0.3

Supplementary Figure S3: Bayesian phylogeny of the partial *L-gene* of Rodentia-associated *UMRV* infecting Tunisian-rodents and sequences on GenBank. The branches colors are represented according to the geographic origin of the sequences as specified in the figure. Groups are identified according to the host species of the *UMRV*. Viruses preceded by an asterisk indicate a spillover event between *Rattus rattus* and other Rodentia species. Values at node points indicate Bayesian posterior probabilities. Virus designations are as follows: Virus name or virus affiliation/typical host/accession number/origin/collection year/host Family. Abbreviations used are detailed in Supplementary Data 4. Two *Respirovirus* sequences (HQ660195 and AB844426) were used as an outgroup.

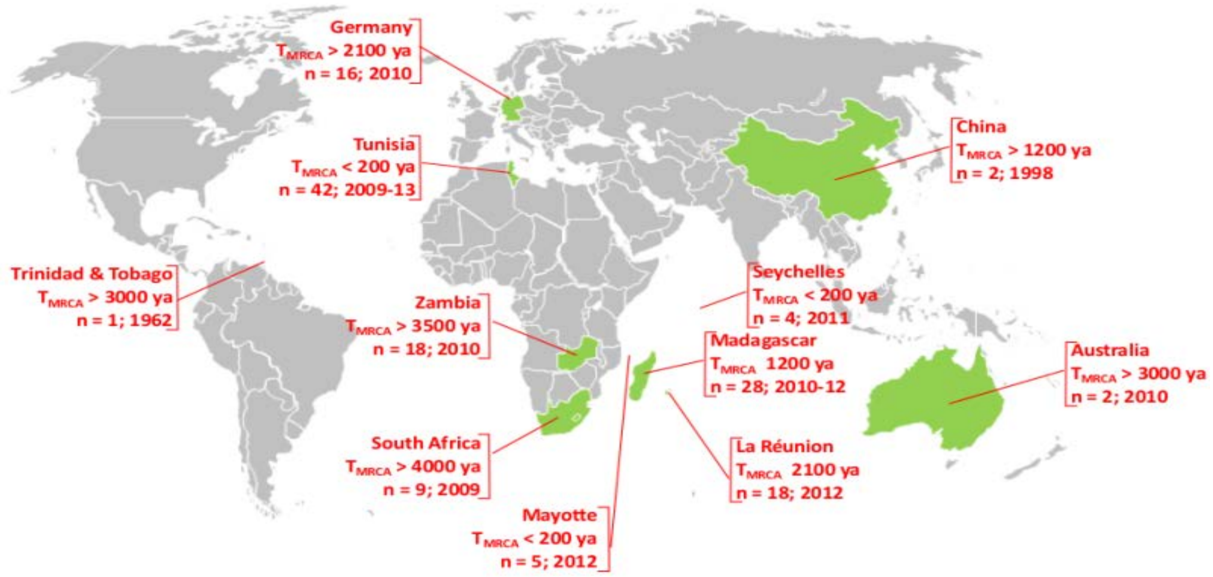

Supplementary Figure 4: Distribution of Rodentia associated *UMRV* isolates and their introduction dates. T_{MRCA}: The most recent common ancestor; n: numbers of *UMRV* isolates and the dates accompanying those numbers indicate the dates of rodent reservoir hosts collection. Map was generated using ArcGIS software (version 10.1; https://www.arcgis.com/features/).

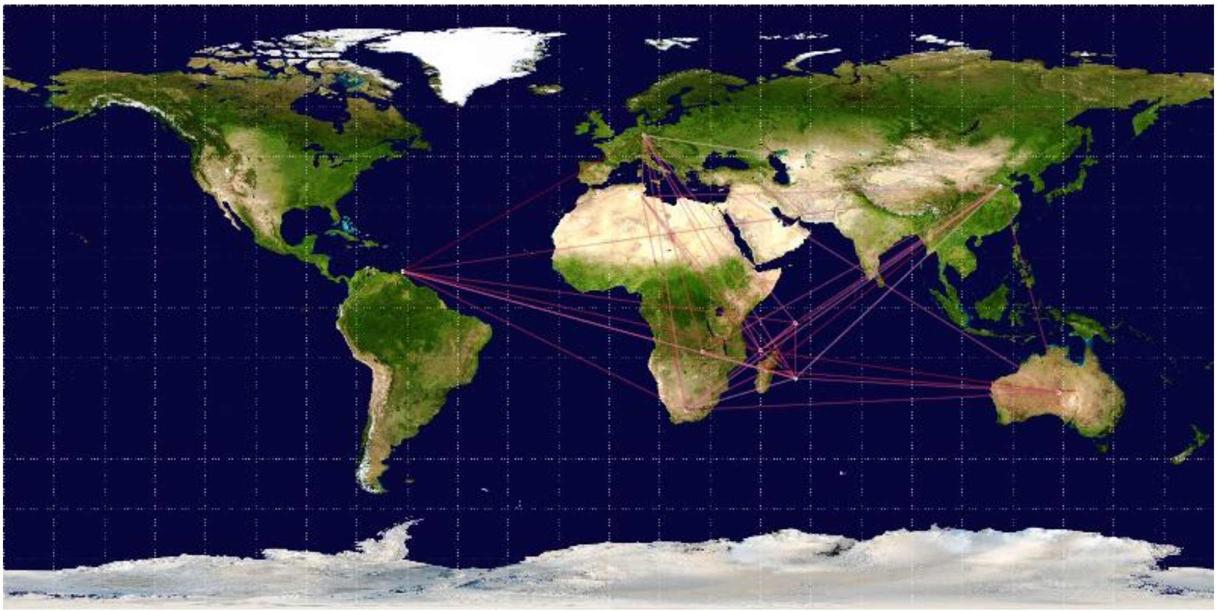

Supplementary Figure 5: The hypothetical worldwide dispersion patterns of Rodentia associated *UMRV* isolates as reconstructed on the basis of significant genetic flow rates and dated phylogeny. Only the rates supported by a Bayes Factor (BF) greater than 3 are shown. The map was reconstructed using SPREAD[1] and visualized using Google Earth (http://earth. google.com). This figure is similar but not identical to the original image, and is therefore for illustrative purposes only.

Supplementary Table S1: Test of substitution saturation for the partial and the full *L-gene* among the *Paramyxoviridae*.

Subsets	NumOTU	Iss	Iss.c Sym	Iss.c Asym
**Partial *L-Gene***	**4**	0,641	0,790	0,758
	**8**	0,656	0,745	0,633
	**16**	0,659	0,707	0,498
	**32**	0,662	0,694	0,366
**Full *L-Gene***	**4**	0,700	0,850	0,841
	**8**	0,700	0,845	0,766
	**16**	0,705	0,831	0,678
	**32**	0,706	0,811	0,563

Iss: index of substitution saturation; Iss.c: critical index of substitution saturation; Sym: symmetrical; Asym: asymmetrical
Subsets corresponding to those used in Supplementary Fig. 1 (for sequence details, see Supplementary Data 3)

Supplementary Table S2. Morphometric and demographic descriptions of rodents from Tunisia: *Psammomys* (*P.*) *obesus*, *Meriones* (*M.*) *shawi*, *Rattus* (*R.*) *rattus* and *Ctenodactylus* (*C.*) *gundi.*

	P. obesus	***M. shawi***	***R. rattus***	***C. gundi***
**Governorates (n (%))**				
Sidi Bouzid	40 (100)	40 (100)	40 (100)	20 (50)
Tataouine	/	/	/	20 (50)
**Sex (n (%))**				
Male	23 (57.5)	15 (37.5)	22 (55)	20 (50)
Female	17 (42.5)	25 (62.5)	18 (45)	20 (50)
**Standard morphometric parameters (Mean±SD)**				
Weight (g)	124±25.20	81.82±26.34	98.12±33.67	224.52±54.85
Ear length (mm)	16.15±1.03	16.4±2.51	21.62±1.68	18.07±1.28
Head and body l ength (mm)	153.15±9.98	138.7±18.04	163.35±24.69	198.1±16.31
Tail length (mm)	128.27±7.86	137.92±14.91	181.42±60.91	28.75±9.87
Hind Feet length (mm)	36.17±1.35	33.75±2.79	33.07±2.55	41.92±2.26

Supplementary Table S3: Viruses used for the estimate of the evolutionary rate change for the partial *L-gene* among the *Measles virus*.

**Virus ID** [a,b]	**Accession numbers**	**Country**	**Collection year**	**Host** [b]
Measles_virus/Human/FJ161211/China/2006	FJ16121	China	2006	*Human*
Measles_virus/ND/HM439386/Sudan/1997	HM439386	Sudan	1997	*ND*
Measles_virus/Human/JF727649/Russia/2010	JF727649	Russia	2010	*Human*
Measles_virus/Human/JF727650/Russia/2010	JF727650	Russia	2010	*Human*
Measles_virus/Human/GQ376026/Japan/1999	GQ376026	Japan	1999	*Human*
Measles_virus/Human/GQ376027/Japan/1999	GQ376027	Japan	1999	*Human*
Measles_virus/ND/AB481088/Japan/1989	AB481088	Japan	1989	*ND*
Measles_virus/ND/AB481087/Japan/1989	AB481087	Japan	1989	*ND*
Measles_virus/Human/KC164757/Italy/2010	KC164757	Italy	2010	*Human*
Measles_virus/ND/KJ018971/Canada/2010	KJ018971	Canada	2010	*ND*
Measles_virus/Human/KJ410048/Germany/2013	KJ410048	Germany	2013	*Human*
Measles_virus/Human/KC164758/Italy/2011	KC164758	Italy	2011	*Human*
Measles_virus/ND/KJ018970/Canada/2010	KJ018970	Canada	2010	*ND*
Measles_virus/ND/KM054581/France/2014	KM054581	France	2014	*ND*
Measles_virus/Human/JN635406/USA/2008	JN635406	USA	2008	*Human*
Measles_virus/Human/JN635410/USA/2003	JN635410	USA	2003	*Human*
Measles_virus/Human/JN635403/USA/2009	JN635403	USA	2009	*Human*
Measles_virus/Human/JN635408/USA/2005	JN635408	USA	2005	*Human*
Measles_virus/Human/JN635402/USA/2009	JN635402	USA	2009	*Human*
Measles_virus/Human/JN635411/USA/2009	JN635411	USA	2009	*Human*
Measles_virus/Human/JN635407/USA/2007	JN635407	USA	2007	*Human*
Measles_virus/Human/JN635404/USA/2009	JN635404	USA	2009	*Human*
Measles_virus/Human/JN635405/USA/2008	JN635405	USA	2008	*Human*
Measles_virus/Human/JN635409/USA/2004	JN635409	USA	2004	*Human*

[a]: Virus name/ typical host/ accession number/origin/collection year
[b]: ND = not defined.

Supplementary Text 1: Comparing partial-*L-Gene* to complete *L-Gene* sequences.

In order to determine if partial *L-gene* sequences can be used to conduct phylogenetic analyses at the level of viral species, we carried out a double phylogenetic analysis using a representative set of the *Paramyxoviridae* containing 179 different sequences and comparison of the associated phylogenetic tree to the one derived from the analysis of the polymerase locus across its full length (8373 base pairs). In Supplementary Fig. 1 we present the two derived trees, which mirror each other regarding to the topology of viral genera and with significant Posterior Bayesian values. There is a notable level of concordance between the two trees at the level of viral species (outlined in color). Seven topological inconsistencies were found, each identified in the figure by a red asterisk, represent less than 4% of the total number of analyzed sequences (n = 7/179). Observed inconsistencies, include *Salem virus* and *Tupaia virus* (Unclassified *Paramyxovirinae*), *Mapuera virus* (Unclassified *Rubulavirus*) and four species of avian paramyxoviruses (APMV-3, -5, -7 and -11) belonging to the genus *Avulavirus*.

Concerning the genus *Morbillivirus*, it is placed after the Rodentia clade (full *L-gene*), or as a sister-clade (partial *L-gene*). In order, to differentiate between these two phylogenetic constructions, we performed statistical analyses of phylogenetic signals by measuring the substitution saturation rate with an entropy-based test implemented in the DAMBE software[2, 3], according to the methodology suggested by these authors. The test consists of measuring an index of critical substitution saturation (Iss.c) assuming either a symmetrical (Iss.cSym) or an asymmetrical (Iss.cAsym) topology. The two-tailed tests establish the critical Iss.c value at which the sequences begin to fail recovery of the true tree for a given data set. Iss > Iss.c indicates that the saturation level is beyond the critical threshold, with a substantial saturation leading to the erosion of the phylogenetic signal. In such cases it is based on a set of sequences not applicable for phylogenetics, whereas conversely an Iss < Iss.c, indicates

relatively low substitution saturation, and associated derived phylogenetic analyses can be used with confidence. The results of this statistical test indicate that the two sets always obtain Iss values close to their respective Iss.c values (assuming a symmetrical topology), while the Iss values are still inferior (Supplementary Table 1). However, assuming an asymmetrical topology, the results are substantially larger for operational taxonomic units Num(OTUs) 16 and 32; the most likely explanation would be a mismatch between the real topology of the *Paramyxoviridae* phylogeny and the one assuming a true asymmetrical tree[2]. The test does not allow a clear discrimination between short or long sequences. However, it demonstrates in both cases, that there is a relatively high substitution saturation (Iss ~ Iss.c). This could be problematic when using long sequences as if we consider an equal saturation process (substitution accumulation), the background noise could be prevailing and reduce the phylogenetic signal. Finally, the seven topological inconsistencies found between the two trees cannot be attributed to either of the trees. It is probable that both phylogenetic constructions are valid. Thus, we consider that the use of partial *L-gene* sequences is a viable alternative for *Paramyxoviridae* phylogenetic reconstruction and, in particular, given that for *UMRV* no complete sequence has been yet reported.

In order to consider phylodynamics in *Paramyxoviridae*, it is necessary to demonstrate that the partial *L-gene* region allows correct extrapolations of driving predictions, which we have already carried out on this viral family from orthologous genes different from the *polymerase gene* (Supplementary Text 2).

It has often been suggested that the use of sequences of a few hundred bases does not provide sufficient information to correctly infer phylogenetic relationships[4]. Even though in certain cases this point is justified, one should be cautious about such generalizations. The verification of the validity of the phylogenetic analyses using short sequences remains an

appropriate approach, particularly when complete sequences are not available[5]. The most common challenge in molecular phylogenetic analyses is the discordance between phylogenies. Even if a simple addition of sequences at a genomic-scale helps clarify different phylogenetic inconsistencies, in some cases maybe insufficient to resolve relationships. Philippe *et al.*[6] support this idea based on two aspects: (i) orthologous genes do not provide the same level of genetic information, owing to the variety of different sources and/or (ii) the loss of phylogenetic signal due to saturation of genetic information by too many substitutions[7, 8].

For PV, gene order may not be the same, i.e. the genus *Respirovirus* as compared to different genera of *Paramyxovirinae* with interchanging *F*- and *G-genes* positions. These differences in the genomic structure probably reflect some internal rearrangement events (duplication/deletion, translocation, etc.) illustrating different evolutionary schemes. Consequently, genomic information may not be considered as an absolute, and hence, genomic level phylogenies for paramyxoviruses may not reflect their true evolutionary trajectory. Besides, in situations when the "background noise" confounds the genuine phylogenetic signal, which is often associated with RNA viruses because of their notably quick evolution, data has to be employed either with the strongest phylogenetic signal, or with shorter sequence lengths[9]. In the case of *Paramyxoviridae*, and particularly for the *UMRV*, recent studies have shown that the short region from the *L Polymerase gene*, namely partial *L-gene*, can provide satisfactory phylogenetic reconstruction at the genus level[10, 11, 12, 13]. As discussed elsewhere by Horreo[14], we consider the partial *L-gene* region as a useful "representative" region of the *Paramyxoviridae* for phylogenetic inference.

Supplementary Text 2: Estimate of the evolutionary rate change for the partial *L-gene* among the *Measles virus*.

As Rodentia PV presented in this study are very close *Morbilli-related* viruses[10, 11, 15] and for some authors probably could be considered as sister genera[13] we used the *Measles virus* (MeV) as a reference for the analysis of evolutionary changes of *UMRV*. Out of 189 complete genomes, 24 MeV partial *L-gene* sequences globally distributed have been selected according to the criterion of the date of collection availability, spanning the years between 1989 and 2014 (Supplementary Table 3). Besides, one might think that only 24 sequences of the MeV may lead to inaccurate estimates; however, to compensate this aspect, the *L-gene* may provide the most reliable evolutionary signal at the family level. The best-fit substitution model was K2 + G, but we used HKY85 + G the nearest close relative best-fit model available in Beauti (Beast package 1.8.2). We used serial samples of MeV for the partial *L-gene* to estimate the rate of nucleotide substitution and the time to the most recent common ancestor (t_{MRCA}), using the Bayesian MCMC method with a coalescent skyline plot method, allowing the analysis of the distribution branch length among collected viruses at different times sampled from millions of trees. We performed a lognormal relaxed molecular clock[16] and a random local clock (RLC)[17], either with a constant population size or a skyline demographic model. The RLC model submits and analyses a succession of different local molecular clocks, each potentially occurring on any branch and extending over a contiguous part of the phylogeny. Runs were carried out with chain lengths of 100-200 millions. The output from Beast was analyzed using the program Tracer 1.6 (http://beast.bio.ed.ac.uk/Tracer). Obtained mean rate was used to the subsequent *UMRV* phylogeographic analysis.

The majority of the set comprising the 145 sequences of *UMRV* felt in a too small time interval, introducing too much bias to determine empirically the evolutionary history and migration patterns of these viruses directly from our data set. This is due to the fact that

different field campaigns to collect samples have all been conducted between 2009 and 2013. However, it is reasonable to assume that determining the evolutionary rate change a virus-related of the same genera or from a virus that proceeds to the same common ancestor could remain a good alternative and a reliable approximation in our case (*L-gene*). Parameter estimates were consistent among models and similar values were obtained for all the analyses. The evolutionary rate of the current circulating MeV for the *L-gene* was estimated to be 3.48 x 10^{-4} substitutions per site per year (95% HPD 3.94 x 10^{-5}, 6.74 x 10^{-4}), and coalescent estimates place its recent emergence at around 1900 (~116 years from 2014, 95% HPD 30.46 to 245.53). The mean evolutionary rate and the t_{MRCAs} derived from the partial *L-gene* were consistent and quietly similar to those already described for other various genes of the current circulating MeV. However, our values were slightly lower than those obtained for the structural *H* and *N* genes[18, 19], but it was not unexpected, being considered that the *polymerase gene* is the most conserved among the *Paramyxoviridae* to maintain crucial structural domains such as multiple functions related to different replicative mechanisms[13, 20]. This molecular clock has been used for the phylogeographic analysis.

References

1. Bielejec, F., Rambaut, A., Suchard, M. A. & Lemey, P. SPREAD: spatial phylogenetic reconstruction of evolutionary dynamics. *Bioinformatics* **27**, 2910-2912 (2011).
2. Xia, X. & Lemey, P. *Assessing Substitution Saturation With DAMBE*. Cambridge University (2009).
3. Xia, X., Xie, Z., Salemi, M., Chen, L. & Wang, Y. An index of substitution saturation and its application. *Mol. Phylogenet. Evol.* **26**, 1-7 (2003).

4. Wani, S. A. *et al.* Whole genome sequence analysis of viruses; moving beyond single/partial gene based phylogenies in context of epidemiology and genetic evolution. *Adv. Anim. Vet. Sci.* **3**, 435-443 (2015).

5. Chernick, A., Godson, D. L., & van der Meer, F. Metadata beyond the sequence enables the phylodynamic inference of bovine viral diarrhea virus type 1a isolates from western Canada. *Infection, Genetics and Evolution* **28**, 367-374 (2014).

6. Philippe, H. *et al.* Resolving difficult phylogenetic questions: why more sequences are not enough. *PLoS Biol.* **9**, e1000602 (2011).

7. Philippe, H., Delsuc, F., Brinkmann, H. & Lartillot, N. Phylogenomics. *Annu. Rev. Ecol. Evol. Syst.* **36**, 541–562 (2005).

8. Townsend, J. P., Su, Z. & Tekle, Y. I. Phylogenetic signal and noise: predicting the power of a data set to resolve phylogeny. *Syst. Biol.* **61**, 835-849 (2012).

9. Jeffroy, O., Brinkmann, H., Delsuc, F. & Philippe, H. Phylogenomics: the beginning of incongruence? *Trends Genet.* **22**, 225-231 (2006).

10. Drexler, J. F. *et al.* Bats host major mammalian paramyxoviruses. *Nat. Commun.* **3**, 796 (2012).

11. Wilkinson, D. *et al.* Highly diverse *Morbillivirus*-related paramyxoviruses in wild fauna of the southwestern Indian Ocean islands: evidence of exchange between introduced and endemic small mammals. *J. Virol.* **88**, 8268-8277 (2014).

12. Melade, J. *et al.* An eco-epidemiological study of *Morbilli*-related paramyxovirus infection in Madagascar bats reveals host-switching as the dominant macro-evolutionary mechanism. *Sci. Rep.* **6**, 23752 (2016).

13. McCarthy, A. J. & Goodman, S. J. Reassessing conflicting evolutionary histories of the Paramyxoviridae and the origins of respiroviruses with Bayesian multigene phylogenies. *Infect. Genet. Evol.* **10**, 97-107 (2010).

14. Horreo, J. L. 'Representative Genes', is it OK to use a small amount of data to obtain a phylogeny that is at least close to the true tree? *J. Evol. Biol.* **25**, 2661-2664 (2012).

15. Sasaki, M. *et al.* Molecular epidemiology of paramyxoviruses in Zambian wild rodents and shrews. *J. Gen. Virol.* **95**, 325-330 (2014).

16. Drummond, A. J., Ho, S. Y., Phillips, M. J. & Rambaut, A. Relaxed phylogenetics and dating with confidence. *PLoS Biol.* **4**, e88 (2006).

17. Drummond, A. J. & Suchard, M. A. Bayesian random local clocks, or one rate to rule them all. *BMC Biol.* **8**, 114 (2010).

18. Pomeroy, L. W., Bjornstad, O. N. & Holmes, E. C. The evolutionary and epidemiological dynamics of the *Paramyxoviridae*. *J. Mol. Evol.* **66**, 98-106 (2008).

19. Furuse, Y., Suzuki, A. & Oshitani, H. Origin of measles virus: divergence from rinderpest virus between the 11th and 12th centuries. *Virol. J.* **7**, 52 (2010).

20. Cox, R. & Plemper, R. K. The paramyxovirus polymerase complex as a target for next-generation anti-paramyxovirus therapeutics. *Front Microbiol.* **6**, 459 (2015).
